# Supplementary material for: Hybridizing salamanders experience accelerated diversification
Source: Sci Rep. 2020 Apr 16;10:6566. doi: 10.1038/s41598-020-63378-w (PMC7162952; doi:10.1038/s41598-020-63378-w)
Supplement: Supplementary file 1 — Supplementary Information. [file 41598_2020_63378_MOESM1_ESM.pdf]

Hybridizing salamanders experience accelerated diversification

Austin H. Patton[1,*], Mark J. Margres[1,2], Brendan Epstein[1,3], Jon Eastman[1], Luke J. Harmon[4], and Andrew Storfer[1]

[1]Washington State University, School of Biological Sciences, Pullman, WA, 99164, USA
[2]Harvard University, Department of Organismic and Evolutionary Biology, Cambridge, MA, 02138, USA
[3]University of Minnesota, College of Biological Sciences, Saint Paul, MN, 55108, USA
[4]University of Idaho, Department of Biological Sciences and IBEST, Moscow, ID, 83844, USA
[*]austin.patton@wsu.edu

Supplementary Materials

Materials and Methods

Assessment of Trait-Dependent Diversification: HiSSE

Through the incorporation of hidden states, the model may be reparameterized to generate a set of competing models, varied in the number of hidden states contained in the model, as well as whether diversification and/or transition rates are allowed to vary among states. Implemented in a likelihood framework, information theoretic approaches (e.g. AIC, AICc) may thus be used to assess fit among competing models. We implemented 14 competing models that varied in the number of hidden states, as well as in the number of unique sets of transition and diversification rates included in the model (Supplementary Table S1). Each of these models were assessed using four datasets described in the manuscript. Briefly, these include 1) all species, 2) only species in sympatry with another species, 3) only plethodontids, and 4) only non-plethodontids. We did not implement HiSSE models assuming equal transition rates among states as the rarity and dispersion of our trait across the tree (Fig. S1) would force the estimation of unrealistically high rates of both speciation and extinction to explain their distribution were transition rates forced to be equal.

By default, HiSSE produces simple transformations of the Speciation Rate (λ) and Extinction Rate (μ). Specifically, it produces rate estimates of the Extinction Fraction ($\varepsilon = \mu \div \lambda$) and Turnover Rate ($\tau = \lambda + \mu$). While the untransformed rate estimates (λ & μ) are conventionally reported, difficulties in obtaining precise estimates of extinction rates in the SSE framework are well documented[63]. However, this may be ameliorated to an extent through the

transformation of rate estimates into ε and τ[61]. Consequently, we report each of the four diversification rates in addition to Net Diversification which is simply λ - μ. Transition rates among character states are reported, although we are less interested in these than we are in estimates for diversification rates.

We choose not to use the semi-parametric FiSSE[112], as this method necessitates symmetric transition rates among character-states. As our character-states exhibit dramatic asymmetry in transition rates, this approach is unsuitable. Further, the DR statistic[113] used as a heuristic for speciation rate is only accurate under a pure-birth model and does not capture the net-diversification rate[114]. Thus, if extinction plays an important role in the diversification process, FiSSE cannot detect it and may be confounded by it.

Additional Datasets

To define sympatry, we needed to collect range polygons, calculate range areas, and calculate areas of intersection for all pairwise combinations of species. Using these range areas and range intersections, we then were able to calculate the percentage of overlap for all pairwise combinations. Range polygons of all available Caudates were downloaded from the IUCN Redlist Webpage[115]. We then converted these polygons to the Mollweide equal area projection, and we then calculated range areas and areas of pairwise intersections using the R packages rgdal v1.2-8[116], PBSmapping v2.70.4[117], and fuzzySim v1.7.9[118]. The resultant matrix was then used to manually calculate percentage overlaps, and only those species sharing at least 10% of their range with another species were retained. Because the *Plethodon glutinosus* group (Figure 1B) has been the subject of extensive taxonomic revision using molecular data (e.g. [39–42]), there exists the distinct possibility that the signature between hybridization and diversification rates in

this group leads to biased inferenced. To account for this potential confounding effect, we re-analyzed the complete tree excluding the *Plethodon glutinosus* group, using both the narrow and broad definitions of hybridization.

Assessing Diversification Rate Differences: All Datasets

In lieu of direct model comparison and reporting parameters under a single model, we adopt the approach of Caetano et al. [65] and report model-averaged parameter estimates. Although the inclusion of hidden states and non-trivial null models greatly improves HiSSE's model rejection properties over related SSE models[61], comparing model-averaged parameter estimates between states enables HiSSE to distinguish between trait-dependent and independent diversification under the most pathological circumstances[65]. Consequently, we report model-averaged parameter estimates for hybridizing and non-hybridizing species. Using this approach, we obtain lineage-specific diversification rates which we then use to conduct an empirical test of diversification rate differences. By making all possible comparisons between non-hybridizing and hybridizing lineages as a ratio (NH/H), we obtain an empirical p-value that communicates the probability that non-hybridizing lineages possess diversification rates that are greater-than or less-than those of hybridizing lineages.

Incomplete taxon-sampling

As implemented by HiSSE, sampling fractions are specified for each state, rather than for taxonomic groups. Importantly, it is assumed that sampling of each state is random; to test whether non-random sampling could bias our results[119], we ask whether clade-specific sampling

fraction predicts prevalence of hybridization. We conduct this test at both the family and genus level (Supplementary Fig. S5), regressing clade-specific sampling fraction against the (sampled) proportion of each clade that is currently hybridizing.

Sensitivity to Phylogenetic Uncertainty

We next tested whether our results are sensitive to phylogenetic uncertainty. Topological uncertainty for the tree used elsewhere in this study[32] was quantified using Shimodaira–Hasegawa-Like implementation of the approximate likelihood-ratio test. Consequently, we sampled 100 trees from the posterior distribution of trees inferred by Jetz and Pyron[120] (Supplementary Fig. S6). These trees were inferred using imputed data for many taxa and so were not used for other analyses herein. Using these 100 trees, we repeated our HiSSE analysis using all species and assuming 20% of species hybridize according to our narrow definition of hybridization. We subsequently 1) summarized model support across replicate analyses (Supplementary Table S15), and 2) calculated the model averaged diversification rates for hybridizing and non-hybridizing taxa from each replicate analysis (Supplementary Fig. S7). This pooled set of model averaged diversification rates was then analyzed using a Welch's two-sample t-test in R.

Results

Incomplete taxon-sampling

Non-random taxonomic sampling has also been shown to undermine the performance of macroevolutionary methods such as those that estimate diversification rates[119]. However, we

find no evidence that clade-specific sampling fraction predicts the frequency of hybridization (Supplementary Fig. S5) in the respective families (Adjusted R^2 = 5.2e-7, P = 1) or genera (Adjusted R^2 = 3.0e-3, P = 0.67).

Sensitivity to Phylogenetic Uncertainty

Our findings appear robust to phylogenetic uncertainty. Using 100 phylogenies drawn from the posterior distribution of Jetz & Pyron[120], a model of trait-independent diversification was never favored (Supplementary Table S15). In other words, trait-dependent diversification was always supported as the best-fit model. Further, as in our main analyses, a HiSSE model was always favored over a simpler BiSSE model. Investigation of model averaged parameter estimates reveals that the mean net-diversification rate of hybridizing salamanders is always greater than those of non-hybridizing salamanders (Supplementary Fig. S7). As with the main analyses, this is driven by concurrent increases in speciation rates and decreases in extinction rates.

Exclusion of *Plethodon glutinosus* group

Reanalysis following exclusion of the *Plethodon glutinosus* group supported our main finding that hybridizing species of salamanders experience heightened diversification rates (Supplementary Fig. S13 & S14, Supplementary Table S17). That is, when analyzed under both the narrow and broad definitions of hybridization, models of trait dependent diversification in which hybridizing salamanders experience heightened rates of diversification were overwhelmingly supported.

Discussion

We believe that the frequency of hybridization in nature is equal to or greater than that represented in the literature due to the substantial effort necessary to document hybridization. Thus, although we suspect that there is a bias in the detection of hybridization towards well studied species in our dataset, many of the species included in our phylogeny have poorly understood ecologies (e.g. Bolitoglossans, Hybobiids). Consequently, we anticipate that the frequency of detection of hybridization in these understudied groups is likely to be equally underestimated for those species included in our phylogeny, as well as for those that are not.

Statistical properties of SSE Methods

Davis et al. [104] documented the potential artifactual consequences of extreme tip-ratio biases in character states. Specifically, they state that "In general, investigators should be cautious using the BiSSE method when one of the binary characters in question is exceedingly rare in their data sets (less than 10%)." As hybridization is observed in ~20% of taxa (tip ratio ~5:1), our dataset thus does not meet their criteria for concern. Further, the tip ratio biases most strongly influence inference of rates of character change followed by extinction rates. As the changes in net diversification in our analyses are most strongly dictated by changes in speciation rather than extinction rates, we have little reason to be concerned that the overall findings of our study are confounded by tip-ratio asymmetries.

Lastly, we are not concerned about the impacts of phylogenetic pseudoreplication in our trait. That is, autapomorphies of rapidly diversifying clades may be spuriously attributed to the rapidity of diversification by SSE methods[112,121]. As our trait is broadly distributed about order Caudata, the potential for phylogenetic pseudoreplication is negligible.

Future Directions

With our data (12 genes), we cannot test the hypothesis that ancient hybridization facilitated the diversification of salamanders through the detection of genealogical discordance at the base of the radiation. The methods best suited to testing this hypothesis, namely the ABBA-BABA class of methods which test for an excess of shared, derived allelic variants using a three-, four- or five-taxon tree (e.g. [122–125]), require genomic datasets larger than our own to have appreciable power[126,127]. Thus, tests of Seehausen's hypotheses are only feasible at the macroevolutionary scale using phylogenomic datasets, an avenue of research we encourage.

References

1. Barton, N. H. & Hewitt, G. M. Analysis of hybrid zones. *Annu. Rev. Ecol. Syst.* **16,** 113–148 (1985).
2. Darwin, C. *On the Origin of Species by Means of Natural Selection. (Murray*. (1859).
3. Fisher, R. *The Genetical Theory of Natural Selection. (Clarendon Press*. (1930).
4. Mayr, E. *Animal species and evolution. (Harvard University Press*. (1963).
5. Futuyma, D. J. On the role of species in anagenesis. *Am. Nat.* **130,** 465–473 (1987).
6. Seehausen, O. Hybridization and adaptive radiation. *Trends Ecol. Evol.* **19,** 198–207 (2004).
7. Grant, P. R. & Grant, B. R. Hybridization of bird species. *Science.* **256,** 193–197 (1992).
8. Seehausen, O., Van Alphen, J. J. & Witte, F. Cichlid fish diversity threatened by eutrophication that curbs sexual selection. *Science.* **277,** 1808–1811 (1997).
9. Turelli, M., Barton, N. H. & Coyne, J. A. Theory and speciation. *Trends Ecol. Evol.* **16,** 330–343 (2001).
10. Buerkle, C. A., Morris, R. J., Asmussen, M. A. & Rieseberg, L. H. The likelihood of homoploid hybrid speciation. *Heredity.* **84,** 441–451 (2000).
11. Dowling, T. E. & Secor, C. L. The role of hybridization and introgression in the diversification of animals. *Annu. Rev. Ecol. Syst.* **28,** 593–619 (1997).
12. Rieseberg, L. H., Archer, M. A. & Wayne, R. K. Transgressive segregation, adaptation and speciation. *Heredity.* **83,** 363 (1999).
13. Soltis, P. S. & Soltis, D. E. The role of hybridization in plant speciation. *Annu. Rev. Plant Biol.* **60,** 561–588 (2009).
14. Coyne, J. A. & Orr, H. A. *Speciation. (Sinauer Associates Sunderland*. (MA, 2004).
15. Francillon-Vieillot, H., Arntzen, J. W. & Geraudie, J. Age, growth and longevity of sympatric *Triturus cristatus*, *T. marmoratus* and their hybrids (Amphibia, Urodela): a

skeletochronological comparison. *J. Herpetol.* **24,** 13 (1990).
16. Arntzen, J. W., Jehle, R., Bardakci, F., Burke, T. & Wallis, G. P. Asymmetric viability of reciprocal-cross hybrids between crested and marbled news (*Triturus cristatus* and *T. marmoratus*). *Evolution.* **63,** 1191–1202 (2009).
17. Marques, D. A., Meier, J. I. & Seehausen, O. A combinatorial view on speciation and adaptive radiation. *Trends Ecol. Evol.* (2019).
18. Mallet, J. Hybrid speciation. *Nature* **446,** 279–83 (2007).
19. Mavárez, J. & Linares, M. Homoploid hybrid speciation in animals. *Mol. Ecol.* **17,** 4181–4185 (2008).
20. Gross, B. & Rieseberg, L. H. The ecological genetics of homoploid hybrid speciation. *J. Hered.* **96,** 241–252 (2005).
21. Schumer, M., Rosenthal, G. G. & Andolfatto, P. How common is homoploid hybrid speciation? *Evolution.* **68,** 1553–1560 (2014).
22. Mavárez, J. *et al.* Speciation by hybridization in *Heliconius* butterflies. *Nature* **441,** 868–871 (2006).
23. Wiens, J. J., Engstrom, T. N. & Chippindale, P. T. Rapid diversification, incomplete isolation, and the "speciation clock" in North American salamanders (genus Plethodon): testing the hybrid swarm hypothesis of rapid radiation. *Evolution.* **60,** 2585–2603 (2006).
24. Keller, I. *et al.* Population genomic signatures of divergent adaptation, gene flow and hybrid speciation in the rapid radiation of Lake Victoria cichlid fishes. *Mol. Ecol.* **22,** 2848–2863 (2013).
25. Meier, J. I. *et al.* Ancient hybridization fuels rapid cichlid fish adaptive radiations. *Nat. Commun.* **8,** 14363 (2017).
26. Grant, P. R. & Grant, B. R. *Darwin's finches on Daphne Major island.* (Princeton University Press, 2014).
27. MacLeod, A. *et al.* Hybridization masks speciation in the evolutionary history of the Galápagos marine iguana. *Proc. R. Soc. B.* **282,** 20150 (2015).
28. Yakimowski, S. B. & Rieseberg, L. H. The role of homoploid hybridization in evolution: a century of studies synthesizing genetics and ecology. *Am. J. Bot.* **101,** 1247–1258 (2014).
29. Seehausen, O. & others. Genomics and the origin of species. *Nat. Rev. Genet.* **15,** 176–192 (2014).
30. Gompert, Z. & Buerkle, C. A. What, if anything, are hybrids: enduring truths and challenges associated with population structure and gene flow. *Evol. Appl.* **9,** 909–923 (2016).
31. University of California, Berkeley. *Species by the numbers.* (2018). at <http://www.amphibiaweb.org/amphibian/speciesnums.html>
32. Pyron, R. A. Biogeographic analysis reveals ancient continental vicariance and recent oceanic dispersal in amphibians. *Syst. Biol.* **63,** 779–797 (2014).
33. Wiens, J. J. Speciation and ecology revisited: phylogenetic niche conservatism and the origin of species. *Evolution.* **58,** 193–197 (2004).
34. Kozak, K. H., Weisrock, D. W. & Larson, A. Rapid lineage accumulation in a non-adaptive radiation: Phylogenetic analysis of diversification rates in eastern North American woodland salamanders (Plethodontidae: Plethodon). *Proc. R. Soc. B Biol. Sci.* **273,** 539–546 (2006).

35. Wake, D. B. Problems with species: patterns and processes of species formation in salamanders. *Ann. Missouri Bot. Gard.* **93,** 8–23 (2006).
36. Vences, M. & Wake, D. B. in *Amphibian Biology, Vol. 6, Systematics* (eds. Heatwole, H. H. & Tyler, M.) 2613–2669 (Surrey Beatty & Sons, Chipping Norton, Australia, 2007).
37. Wake, D. B. What Salamanders have Taught Us about Evolution. (2009). doi:10.1146/annurev.ecolsys.39.110707.173552
38. Blankers, T., Adams, D. C. & Wiens, J. J. Ecological radiation with limited morphological diversification in salamanders. *J. Evol. Biol.* **25,** 634–646 (2012).
39. Highton, R. Speciation in eastern North American salamanders of the genus *Plethodon. Annu. Rev. Ecol. Syst.* **26,** 579–600 (1995).
40. Highton, R. Geographic protein variation and speciation in the *Plethodon dorsalis* complex. *Herpetologica* **1997,** 345–356 (1997).
41. Highton, R. Geographic protein variation and speciation in the salamanders of the Plethodon cinereus group with the description of two new species. *Herpetologica* **1999,** 43–90 (1999).
42. Highton, R. & Peabody, R. in *The Biology of Plethodontid Salamanders.* (eds. Bruce, R. C., Jaeger, R. G. & Houck, L. D.) 31–93 (Kluwer Academic/Plenum Publishers, 2000).
43. Wake, D. B. & Jockusch, E. L. in *The Biology of Plethodontid Salamanders* 95–119 (Springer US, 2000). doi:10.1007/978-1-4615-4255-1_4
44. Patton, A. *et al.* A New Green Salamander in the Southern Appalachians: Evolutionary History of Aneides aeneus and Implications for Management and Conservation with the Description of a Cryptic Microendemic Species. *Copeia* **107,** (2019).
45. Rundell, R. J. & Price, T. D. Adaptive radiation, nonadaptive radiation, ecological speciation and nonecological speciation. *Trends Ecol. Evol.* **24,** 394–399 (2009).
46. Czekanski-Moir, J. E. & Rundell, R. J. The ecology of nonecological speciation and nonadaptive radiations. *Trends Ecol. Evol.* (2019).
47. Garcia-Paris, M., Good, D. A., Parra-Olea, G. & Wake, D. B. Biodiversity of Costa Rican salamanders: Implications of high levels of genetic differentiation and phylogeographic structure for species formation. *Proc. Natl. Acad. Sci.* **97,** 1640–1647 (2000).
48. Parra-Olea, G. & Wake, D. B. Extreme morphological and ecological homoplasy in tropical salamanders. *Proc. Natl. Acad. Sci. U. S. A.* **98,** 7888–7891 (2001).
49. Vieites, D. R., Min, M. S. & Wake, D. B. Rapid diversification and dispersal during periods of global warming by plethodontid salamanders. *Proc. Natl. Acad. Sci. U. S. A.* **104,** 19903–19907 (2007).
50. Parra-Olea, G., Windfield, J. C., Velo-Antón, G. & Zamudio, K. R. Isolation in habitat refugia promotes rapid diversification in a montane tropical salamander. *J. Biogeogr.* **39,** 353–370 (2012).
51. Bryson, R. W. *et al.* Phylogenomic insights into the diversification of salamanders in the *Isthmura bellii* group across the Mexican highlands. *Mol. Phylogenetics Evol.* **125,** 78–84 (2018)
52. Gillespie, R. G. *et al.* Comparing Adaptive Radiations Across Space, Time, and Taxa. *J. Hered.* **89154,** 14853 (2020).
53. Stamatakis, A. RAxML-VI-HPC: maximum likelihood-based phylogenetic analyses with thousands of taxa and mixed models. *Bioinformatics* **22,** 2688–2690 (2006).

54. Smith, S. A. & O'meara, B. C. treePL: divergence time estimation using penalized likelihood for large phylogenies. *Bioinformatics* **28,** 2689–2690 (2012).
55. Paradis, E., Claude, J. & Strimmer, K. APE: analyses of phylogenetics and evolution in R language. *Bioinformatics* **20,** 289–290 (2004).
56. Liu, F. G. R., Moler, P. E. & Miyamoto, M. M. Phylogeography of the salamander genus *Pseudobranchus* in the southeastern United States. *Mol. Phylogenet. Evol.* **39,** 149–159 (2006).
57. Fu, J. & Zeng, X. How many species are in the genus *Batrachuperus*? A phylogeographical analysis of the stream salamanders (family *Hynobiidae*) from southwestern China. *Mol. Ecol.* **17,** 1469–1488 (2008).
58. Yoshikawa, N., Matsui, M., Nishikawa, K., Misawa, Y. & Tanabe, S. Allozymic Variation in the Japanese Clawed Salamander, Onychodactylus japonicus (Amphibia: Caudata: *Hynobiidae*), with Special Reference to the Presence of Two Sympatric Genetic Types. *Zoolog. Sci.* **27,** 33–40 (2010).
59. Yoshikawa, N., Matsui, M. & Nishikawa, K. Genetic Structure and Cryptic Diversity of *Onychodactylus japonicus* (Amphibia, Caudata, *Hynobiidae*) in Northeastern Honshu, Japan, as Revealed by Allozymic Analysis. *Zoolog. Sci.* **29,** 229–237 (2012).
60. Yoshikawa, N. & Matsui, M. Two new Salamanders of the genus *Onychodactylus* from Eastern Honshu, Japan (Amphibia, Caudata, *Hynobiidae*). (2014). doi:10.11646/zootaxa.3866.1.3
61. Beaulieu, J. M. & O'Meara, B. C. Detecting hidden diversification shifts in models of trait dependent speciation and extinction. *Syst. Biol.* **65,** 583–601 (2016).
62. Stadler, T. How can we improve accuracy of macroevolutionary rate estimates? *Syst. Biol.* **62,** 321–329 (2013).
63. Beaulieu, J. M. & O'Meara, B. C. Extinction can be estimated from moderately sized molecular phylogenies. **69,** 1036–1043 (2015).
64. Rabosky, D. L. & Goldberg, E. E. Model inadequacy and mistaken inferences of trait-dependent speciation. *Syst. Biol.* **64,** 340–55 (2015).
65. Caetano, D. S., O'Meara, B. C. & Beaulieu, J. M. Hidden state models improve state-dependent diversification approaches, including biogeographical models. *Evolution (N. Y).* (2018).
66. Barraclough, T. G., Harvey, P. H. & Nee, S. Rate of rbcL gene sequence evolution and species diversification in flowering plants (angiosperms). Proceedings of the Royal Society of London. *Ser. B. Biol. Sci.* **263,** 589–591 (1996).
67. R Core Team. R: A Language and Environment for Statistical Computing. (2018). at <https://www.r-project.org/>
68. Hewitt, G. M. Some genetic consequences of ice ages, and their role in divergence and speciation. *Biol. J. Linn. Soc.* **58,** 247–276 (1996).
69. Kozak, K. H., Blaine, R. A. & Larson, A. Gene lineages and eastern North American palaeodrainage basins: phylogeography and speciation in salamanders of the *Eurycea bislineata* species complex. *Mol. Ecol.* **15,** 191–207 (2006).
70. Shepard, D. B. & Burbrink, F. T. Phylogeographic and demographic effects of Pleistocene climatic fluctuations in a montane salamander, *Plethodon fourchensis*. *Mol. Ecol.* **18,** 2243–2262 (2009).

71. Walls, S. C. The role of climate in the dynamics of a hybrid zone in Appalachian salamanders. *Glob. Chang. Biol.* **15,** 1903–1910 (2009).
72. Rice, W. R. & Hostert, E. E. Laboratory experiments on speciation: what have we learned in 40 years? **47,** 1637–1653 (1993).
73. Servedio, M. R. & Noor, M. A. The role of reinforcement in speciation: theory and data. *Annu. Rev. Ecol. Evol. Syst.* **34,** 339–364 (2003).
74. Matute, D. R. & Ortiz-Barrientos, D. Speciation: The strength of natural selection driving reinforcement. *Curr. Biol.* **24,** 955–957 (2014).
75. Zamudio, K. R. & Wieczorek, A. M. Fine-scale spatial genetic structure and dispersal among spotted salamander (*Ambystoma maculatum*) breeding populations. *Mol. Ecol.* **16,** 257–274 (2007).
76. Emel, S. L. & Storfer, A. A decade of amphibian population genetic studies: synthesis and recommendations. *Conserv. Genet.* **13,** 1685–1689 (2012).
77. Meier, J. I. *et al.* The coincidence of ecological opportunity with hybridization explains rapid adaptive radiation in Lake Mweru cichlid fishes. *Nat. Commun.* **10,** (2019).
78. Rico, C. & Turner, G. F. Extreme microallopatric divergence in a cichlid species from Lake Malawi. *Mol. Ecol.* **11,** 1585–1590 (2002).
79. Church, S. A., Kraus, J. M., Mitchell, J. C., Church, D. R. & Taylor, D. R. Evidence for Multiple Pleistocene Refugia in the Postglacial Expansion of the Eastern Tiger salamander, Ambystoma Tigrinum Tigrinum. *Evolution (N. Y).* **57,** 372–383 (2003).
80. Crespi, E. J., Rissler, L. J. & Browne, R. A. Testing Pleistocene refugia theory: phylogeographical analysis of Desmognathus wrighti, a high-elevation salamander in the southern Appalachians. *Mol. Ecol.* **12,** 969–984 (2003).
81. Steele, C. A. & Storfer, A. Phylogeographic incongruence of codistributed amphibian species based on small differences in geographic distribution. *Mol. Phylogenet. Evol.* **43,** 468–479 (2007).
82. Zamudio, K. R. & Savage, W. K. Historical Isolation, Range Expansion, and Secondary Contact of Two Highly Divergent Mitochondrial Lineages in Spotted Salamanders (Ambystoma maculatum). *Evolution (N. Y).* **57,** 1631–1652 (2003).
83. Alexandrino, J., Froufe, E., Arntzen, J. W. & Ferrand, N. Genetic subdivision, glacial refugia and postglacial recolonization in the golden-striped salamander, *Chioglossa lusitanica* (Amphibia: Urodela). *Mol. Ecol.* **9,** 771–781 (2000).
84. Steinfartz, S., Veith, M. & Tautz, D. Mitochondrial sequence analysis of *Salamandra* taxa suggests old splits of major lineages and postglacial recolonizations of Central Europe from distinct source populations of Salamandra salamandra. *Mol. Ecol.* **9,** 397–410 (2000).
85. Mattoccia, M., Marta, S., Romano, A. & Sbordoni, V. Phylogeography of an Italian endemic salamander (genus *Salamandrina*): glacial refugia, postglacial expansions, and secondary contact. *Biol. J. Linn. Soc.* **104,** 903–992 (2011).
86. Kozak, K. H. & Wiens, J. J. Niche conservatism drives elevational diversity patterns in Appalachian salamanders. *Am. Nat.* **176,** 40–54 (2010).
87. Parmesan, C. & Yohe, G. A globally coherent fingerprint of climate change impacts across natural systems. *Nature* **421,** 37–42 (2003).
88. Root, T. L., Hall, P., K. R., S. & S. H. Rosenzweig C. & Pounds, J. A. Fingerprints of

global warming on wild animals and plants. *Nature* **421,** 57–60 (2003).

89. Kelly, A. E. & Goulden, M. L. Rapid shifts in plant distribution with recent climate change. *Proc. Natl. Acad. Sci.* **105,** 11823–11826 (2008).

90. Chen, I. C., Hill, J. K., Ohlemüller, R., Roy, D. B. & Thomas, C. D. Rapid range shifts of species associated with high levels of climate warming. *Science.* **333,** 1024–1026 (2011).

91. Elsen, P. R. & Tingley, M. W. Global mountain topography and the fate of montane species under climate change. *Nat. Clim. Chang.* **5,** 772–776 (2015).

92. Garroway, C. J. *et al.* Climate change induced hybridization in flying squirrels. *Glob. Chang. Biol.* **16,** 113–121 (2010).

93. Hoffmann, A. A. & Sgrò, C. M. Climate change and evolutionary adaptation. *Nature* **470,** 479–85 (2011).

94. Chunco, A. J. Hybridization in a warmer world. *Ecol. Evol.* **4,** 2019–2031 (2014).

95. McQuillan, M. A. & Rice, A. M. Differential effects of climate and species interactions on range limits at a hybrid zone: potential direct and indirect impacts of climate change. *Ecol. Evol.* **5,** 5120–5137 (2015).

96. Pereira, R. J., Martínez-Solano, I. & Buckley, D. Hybridization during altitudinal range shifts: nuclear introgression leads to extensive cyto-nuclear discordance in the fire salamander. *Mol. Ecol.* **25,** 1551–1565 (2016).

97. Seehausen, O. Conservation: losing biodiversity by reverse speciation. *Curr. Biol.* **16,** 334-R337 (2006).

98. Seehausen, O., Takimoto, G., Roy, D. & Jokela, J. Speciation reversal and biodiversity dynamics with hybridization in changing environments. *Mol. Ecol.* **17,** 30–44 (2008).

99. Vonlanthen, P. & others. Eutrophication causes speciation reversal in whitefish adaptive radiations. *Nature* **482,** 357–362 (2012).

100. Hoskin, C. J., Higgie, M., McDonald, K. R. & Moritz, C. Reinforcement drives rapid allopatric speciation. *Nature* **437,** 1353–1356 (2005).

101. Pfennig, K. S. Reinforcement as an initiator of population divergence and speciation. *Curr. Zool.* **62,** 145–154 (2016).

102. Arnold, S. J., Reagan, N. L. & Verrell., P. A. Reproductive isolation and speciation in plethodontid salamanders. *Herpetologica* **49,** 216–228 (1993).

103. Reagan, N. L. *Evolution of sexual isolation in salamanders of the genus Plethodon (Doctoral dissertation)*. University of Chicago. (1992).

104. Davis, M. P., Midford, P. E. & Maddison, W. Exploring power and parameter estimation of the BiSSE method for analyzing species diversification. *BMC Evol. Biol.* **13,** 38 (2013).

105. Freyman, W. A. & Höhna, S. Stochastic character mapping of state-dependent Diversification reveals the tempo of evolutionary decline in self-compatible *Onagraceae* lineages. *Syst. Biol.* **68,** 505–519 (2019).

106. Rabosky, D. L. Extinction rates should not be estimated from molecular phylogenies. *Evolution.* **64,** 1816–1824 (2010).

107. Rabosky, D. L. Challenges in the estimation of extinction from molecular phylogenies: a response to Beaulieu and O'Meara. *Evolution.* **70,** 218–228 (2016).

108. Kearney, M. Hybridization, glaciation and geographical parthenogenesis. *Trends Ecol. Evol.* **20,** 495–502 (2005).

109. Willis, B. L., van Oppen, M. J., Miller, D. J., Vollmer, S. V & Ayre, D. J. The role of

hybridization in the evolution of reef corals. *Annu. Rev. Ecol. Evol. Syst* **37,** 489–517 (2006).
110. Schmid, M., Evans, B. J. & Bogart, J. P. Polyploidy in Amphibia. *Cytogenetic and Genome Research* **145,** 315–330 (2015).
111. Runemark, A., Vallejo-Marin, M. & Meier, J. I. Eukaryote hybrid genomes. *PLOS Genet.* **15,** e1008404 (2019).
112. Rabosky, D. L. & Goldberg, E. E. FiSSE: A simple nonparametric test for the effects of a binary character on lineage diversification rates. *Evolution* **17,** 1432–1442 (2017).
113. Jetz, W., Thomas, G., Joy, J., Hartmann, J. & Mooers, A. The global diversity of birds in space and time. *Nature* **491,** 444–448 (2012).
114. Belmaker, J. & Jetz, W. Relative roles of ecological and energetic constraints, diversification rates and region history on global species richness gradients. *Ecol. Lett.* **18,** 563–571 (2015).
115. *Spatial data download*. (2017). at <http://www.iucnredlist.org/technical-documents/spatial-data/>
116. Bivand, R., Keitt, T. & Rowlingson, B. rgdal: Bindings for the ‘geospatial’ data abstraction library. *R Packag. version* **1,** 2–13 (2017).
117. Schnute, J. T., Boers, N. & Haigh, R. PBSmapping: Mapping fisheries data and spatial analysis tools. (2017).
118. Barbosa, A. M. fuzzysim: applying fuzzy logic to binary similarity indices in ecology. *Methods Ecol. Evol.* **6,** 853–858 (2015).
119. Hua, X. & Lanfear, R. The influence of non-random species sampling on macroevolutionary and macroecological inference from phylogenies. *Methods Ecol. Evol.* **9,** 1353–1362 (2018).
120. Jetz, W. & Pyron, R. A. The interplay of past diversification and evolutionary isolation with present imperilment across the amphibian tree of life. *Nat. Ecol. Evol.* **2,** 850 (2018).
121. Maddison, W. P. & FitzJohn, R. G. The unsolved challenge to phylogenetic correlation tests for categorical characters. *Syst. Biol.* **64,** 127–136 (2014).
122. Hahn, M. W. & Hibbins, M. S. A Three-Sample Test for Introgression. *Mol. Biol. Evol.* **36,** 2878–2882 (2019).
123. Green, R. E. & others. A draft sequence of the Neandertal genome. *Science.* **328,** 710–722 (2010).
124. Durand, E. Y., Patterson, N., Reich, D. & Slatkin, M. Testing for ancient admixture between closely related populations. *Mol. Biol. Evol.* **28,** 2239–2252 (2011).
125. Pease, J. B. & Hahn, M. W. Detection and polarization of introgression in a five-taxon phylogeny. *Syst. Biol.* **64,** 651–662 (2015).
126. Martin, S. H., Davey, J. W. & Jiggins, C. D. Evaluating the use of ABBA–BABA statistics to locate introgressed loci. *Mol. Biol. Evol.* **32,** 244–257 (2014).
127. Zheng, Y. & Janke, A. Gene flow analysis method, the D-statistic, is robust in a wide parameter space. *BMC Bioinformatics* **19,** 10 (2018).
128. Fukumoto, S., Ushimaru, A. & Minamoto, T. A basin-scale application of environmental DNA assessment for rare endemic species and closely related exotic species in rivers: a case study of giant salamanders in Japan. *J. Appl. Ecol.* **52,** 358–365 (2015).

129. Malyarchuk, B. A., Derenko, M. V & Denisova, G. A. Phylogenetic relationships among Asiatic salamanders of the genus *Salamandrella* based on variability of nuclear genes. *Russ. J. Genet.* **51,** 91–97 (2015).

130. Kawamura, T. Interspecific hybrids between *Hynobius nigrescens* and *Hynobius tokyoensis*. *J. Fac. Sci. Hokkaido Univ.* **13,** 248–252 (1957).

131. Steele, C. A., Baumsteiger, J. & Storfer, A. Polymorphic tetranucleotide microsatellites for Cope's giant salamander (*Dicamptodon copei*) and Pacific giant salamander (*Dicamptodon tenebrosus*). *Mol. Ecol. Resour.* **8,** 1071–1073 (2008).

132. Bogart, J. P., Bi, K., Fu, J., Noble, D. W. A. & Niedzwiecki, J. Unisexual salamanders (genus *Ambystoma*) present a new reproductive mode for eukaryotes. *Genome* **50,** 119–136 (2007).

133. Eastman, J. M., Niedzwiecki, J. N., Nadler, B. P. & Storfer, A. Duration and consistency of historical selection are correlated with adaptive trait evolution in the streamside salamander, *Ambystoma barbouri*. *Evolution.* **43,** 468–479 (2009).

134. Fitzpatrick, B. M. & Shaffer, H. B. Hybrid vigor between native and introduced salamanders raises new challenges for conservation. *Proc. Natl. Acad. Sci.* **104,** 15793–15798 (2007).

135. Weisrock, D. W., Shaffer, H. B., Storz, B. L., Storz, S. R. & Voss, S. R. Multiple nuclear gene sequences identify phylogenetic species boundaries in the rapidly radiating clade of Mexican ambystomatid salamanders. *Mol. Ecol.* **15,** 2489–2503 (2006).

136. Canestrelli, D., Bisconti, R. & Nascetti, G. Extensive unidirectional introgression between two salamander lineages of ancient divergence and its evolutionary implications. *Sci. Rep.* **4,** 6516 (2014).

137. Hauswaldt, J. S., Angelini, C., Pollok, A. & Steinfartz, S. Hybridization of two ancient salamander lineages: molecular evidence for endemic spectacled salamanders on the Apennine peninsula. *J. Zool.* **284,** 248–256 (2011).

138. Veith M., *et al.* Cracking the nut: Geographical adjacency of sister taxa supports vicariance in a polytomic salamander clade in the absence of node support. *Mol. Phylogenet. Evol.* **47,** 916–931 (2008).

139. Johannesen, J. *et al.* Distortion of symmetrical introgression in a hybrid zone: evidence for locus-specific selection and uni-directional range expansion. *J. Evol. Biol.* **19,** 705–716 (2006).

140. Escoriza, D., Gutiérrez-Rodríguez, J., Hassine, J. B. & Martínez-Solano, I. Genetic assessment of the threatened microendemic *Pleurodeles poireti* (Caudata, Salamandridae), with molecular evidence for hybridization with *Pleurodeles* nebulosus. *Conserv. Genet.* **17,** 1445–1458 (2016).

141. Phimmachak, S., Aowphol, A. & Stuart, B. L. Morphological and molecular variation in *Tylototriton* (Caudata: Salamandridae) in Laos, with description of a new species. *Zootaxa* **4006,** 285–310 (2015).

142. Davis, W. C. & Twitty, V. C. Courtship behavior and reproductive isolation in the species of *Taricha* (Amphibia, Caudata). *Copeia* **1964,** 601–610 (1964).

143. Hedgecock, D. & Ayala, F. J. Evolutionary divergence in the genus *Taricha* (*Salamandridae*). *Copeia* **1974,** 738–747 (1974).

144. Kuchta, S. R. & Tan, A. M. Isolation by distance and post-glacial range expansion in the rough-skinned newt, *Taricha granulosa*. *Mol. Ecol.* **14,** 225–244 (2005).
145. Kuchta, S. R. & Tan, A. M. Limited genetic variation across the range of the red-bellied newt, *Taricha rivularis*. *J. Herpetol.* **40,** 561–565 (2006).
146. Kuchta, S. R. Contact zones and species limits: hybridization between lineages of the California Newt, *Taricha torosa* in the southern Sierra Nevada. *Herpetologica* **63,** 332–350 (2007).
147. Johanet, A., Secondi, J. & Lemaire, C. Widespread introgression does not leak into allotopy in a broad sympatric zone. *Heredity*. **106,** 962 (2011).
148. Babik, W. *et al.* Phylogeography of two European newt species -- discordance between mtDNA and morphology. *Mol. Ecol.* **14,** 2475–2491 (2005).
149. van Riemsdijk, I. *et al.* The Near East as a cradle of biodiversity: a phylogeography of banded newts (genus *Ommatotriton*) reveals extensive inter-and intraspecific genetic differentiation. *Mol. Phylogenet. Evol.* **114,** 73–81 (2017).
150. Themudo, G., Nieman, A. M. & Arntzen, J. W. Is dispersal guided by the environment? A comparison of interspecific gene flow estimates among differentiated regions of a newt hybrid zone. *Mol. Ecol.* **21,** 5324–5335 (2012).
151. Schoorl, J. & Zuiderwijk, A. Ecological isolation in *Triturus cristatus* and *Triturus marmoratus* (Amphibia: Salamandridae). *Amphibia-Reptilia* **1,** 235–252 (1981).
152. Brede, E. G., Thorpe, R. S., Arntzen, J. W. & Langton, T. E. S. A morphometric study of a hybrid newt population (*Triturus cristatus*/*T. carnifex*): Beam Brook Nurseries. Surrey, UK. *Biol. J. of the Linn. Soc.* **70,** 685-295 (2000).
153. Wielstra, B. & Arntzen, J. W. Postglacial species displacement in *Triturus* newts deduced from asymmetrically introgressed mitochondrial DNA and ecological niche models. *BMC Evol. Biol.* **12,** 161 (2012).
154. Nelson, S. K., Niemiller, M. L. & Fitzpatrick, B. M. Co-occurrence and hybridization between *Necturus maculosus* and a heretofore unknown *Necturus* in the Southern Appalachians. *J. Herpetol.* **51,** 559–566 (2017).
155. Bonett, R. M., Chippindale, P. T., Moler, P. E., Van Devender, R. W. & Wake, D. B. Evolution of gigantism in amphiumid salamanders. *PLoSONE* **4,** (2009).
156. Nascetti, G., Cimmaruta, R., Lanza, B. & Bullini, L. Molecular taxonomy of European plethodontid salamanders (genus *Hydromantes*). *J. Herpetol.* **30,** 161–183 (1996).
157. Tilley, S. G. Review of: Morphologic and Genetic Studies of the European Plethodontid Salamanders: Taxonomic Inferences (Genus *Hydromantes*) by Benedetto Lanza, Vincenzo Caputo, Giuseppe Nascetti, and Luciano Bullini. *Copeia* **1998,** 526–528 (1998).
158. Jackman, T. R. Molecular and historical evidence for the introduction of clouded salamanders (genus *Aneides*) to Vancouver Island, British Columbia, Canada, from California. *Can. J. Zool.* **76,** 1570–1580 (1998).
159. Kozak, K. H. Sexual isolation and courtship behavior in salamanders of the *Eurycea bislineata* species complex, with comments on the evolution of the mental gland and pheromone delivery behavior in the Plethodontidae. *Southeast. Nat.* **2,** 281–292 (2003).
160. Bonett, R. M. Analysis of the contact zone between the dusky salamanders *Desmognathus fuscus fuscus* and *Desmognathus fuscus conanti* (Caudata: Plethodontidae). *Copeia* **105,** 344–355 (2002).

161. Highton, R., *et al.* Concurrent speciation in the eastern woodland salamanders (genus Plethodon): DNA sequences of the complete albumin nuclear and partial mitochondrial 12s genes. *Mol. Phyl. and Evol.* **63**, 278-290. (2012).
162. Highton, R. Geographic protein variation and speciation in the salamanders of the *Plethodon cinereus* group with the description of two new species. *Herpetologica* **1999,** 43–90 (1999).
163. Highton, R. Geographic protein variation and speciation in the *Plethodon* dorsalis complex. *Herpetologica* **1997,** 345–356 (1997).
164. Highton, R. Speciation in eastern North American salamanders of the genus *Plethodon. Annu. Rev. Ecol. Syst.* **26,** 579–600 (1995).
165. Highton, R. & Peabody, R. in *The Biology of Plethodontid Salamanders.* (eds. Bruce, R. C., Jaeger, R. G. & Houck, L. D.) 31–93 (Kluwer Academic/Plenum Publishers, 2000).
166. Niemiller, M. L., Fitzpatrick, B. M. & Miller, B. T. Recent divergence with gene flow in Tennessee cave salamanders (Plethodontidae: *Gyrinophilus*) inferred from gene genealogies. *Mol. Ecol.* **17,** 2258–2275 (2008).
167. Kuchta, S. R., Haughey, M., Wynn, A. H., Jacobs, J. F. & Highton, R. Ancient river systems and phylogeographical structure in the spring salamander, *Gyrinophilus porphyriticus*. *J. Biogeogr.* **43,** 639–652 (2016).
168. Smith, C. C. Problem of the Hybridization of the Red Cave Salamander, *Eurycea lucifuga* (Raf), and the Long-Tailed Salamander, *Eurycea longicauda melanopleura* (Green). *J. Ark. Acad. Sci.* **18,** 59–62 (1964).
169. Sweet, S. S. Secondary contact and hybridisation in the Texas cave salamanders *Eurycea neotenes* and *E. tridentifera*. *Copeia* **1984,** 428–441 (1984).
170. Guttman, S. I. & Karlin, A. A. Hybridisation of cryptic species of two-lined salamanders. (*Eurycea bislineata* complex). *Copeia* **1986,** 96–108 (1986).
171. Kozak, K. H. & Montanucci, R. R. Genetic variation across a contact zone between montane and lowland forms of the Two-lined salamander (*Eurycea bislineata*) species complex: a test of species limits. *Copeia* **2001,** 25–34 (2001).
172. Bendik, N. F., Meik, J. M., Gluesenkamp, A. G., Roelke, C. E. & Chippindale, P. T. Biogeography, phylogeny, and morphological evolution of central Texas cave and spring salamanders. *BMC Evol. Biol.* **13,** 201 (2013).
173. Jockusch, E. L., Martinez-Solano, I., Hansen, R. W. & Wake, D. B. Morphological and molecular diversification of slender salamanders (Caudata: Plethodontidae: *Batrachoseps*) in the southern Sierra Nevada of California with descriptions of two new species. *Zootaxa* **3190,** 30 (2012).
174. Jockusch, E. L. & Wake, D. B. Falling apart and merging: diversification of slender salamanders (Plethodontidae: *Batrachoseps*) in the American West. *Biol. J. Linn. Soc.* **76,** 361–391 (2002).
175. Jockusch, E. L., Yanev, K. P. and Wake, D. B. Molecular phylogenetic analysis of slender salamanders, genus Batrachoseps (Amphibia: *Plethodontidae*), from central coastal California with descriptions of four new species. *Herp. Mono.* 54-99. (2001).
176. Wake, D. B. & Lynch, J. F. Evolutionary relationships among Central American salamanders of the *Bolitoglossa franklini* group, with a description of a new species from Guatemala. *Herpetologica* **38,** 257–272 (1982).

177. Wake, D. B., Yang, S. Y. & Papenfuss, T. J. Natural hybridization and its evolutionary implications in Guatemalan plethodontid salamanders of the genus *Bolitoglossa*. *Herpetologica* **1980,** 335–345 (1980).

Supplementary Figures/Tables

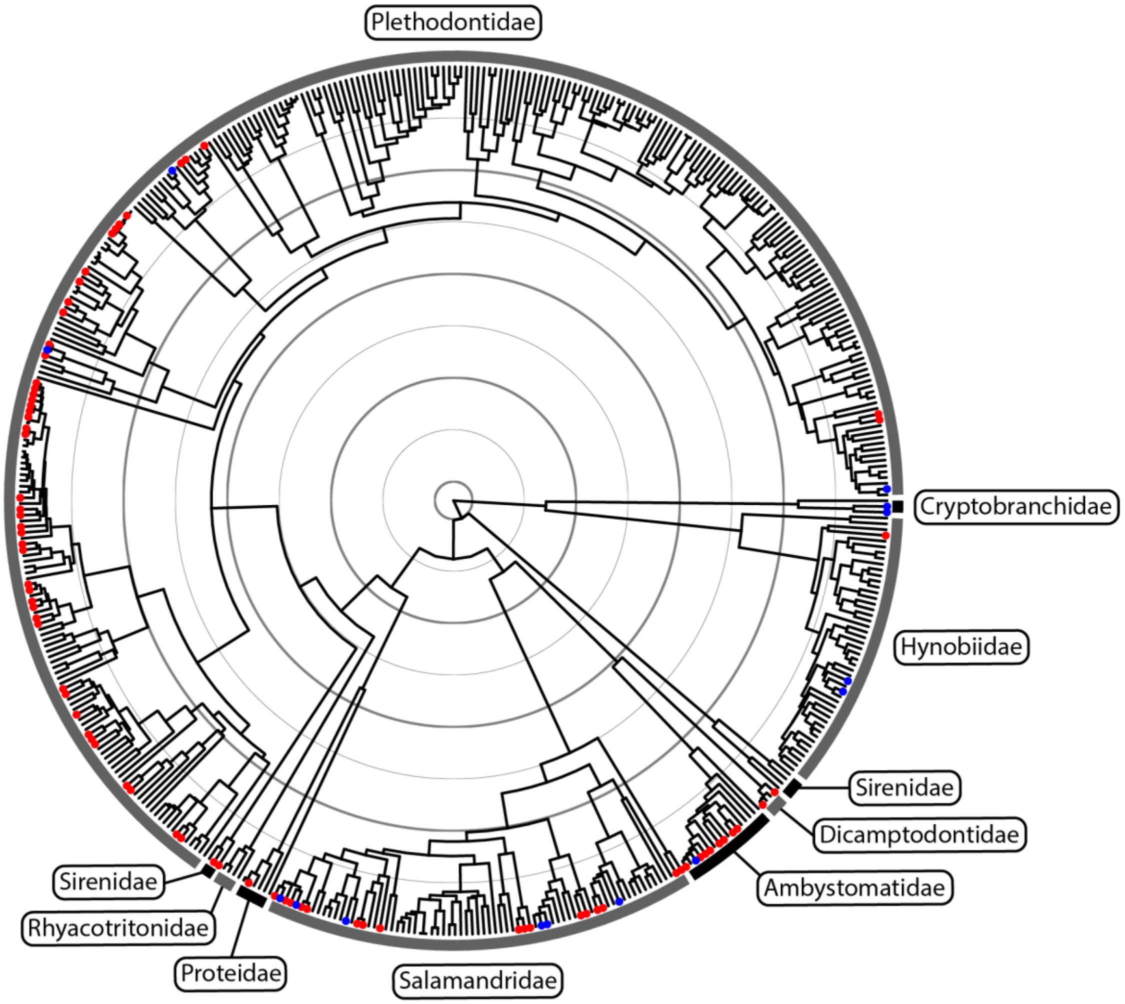

Figure S1. Phylogeny used in analysis of complete dataset. Salamanders were pruned from the time calibrated tree of Pyron (2014). Species observed to hybridize in nature according to the narrow dataset have red circles at the tips. Additional species included in the broad dataset are indicated by blue circles. Families are shown according to the external broken circle, each labeled with their respective names. Each internal circle corresponds to 25 million years.

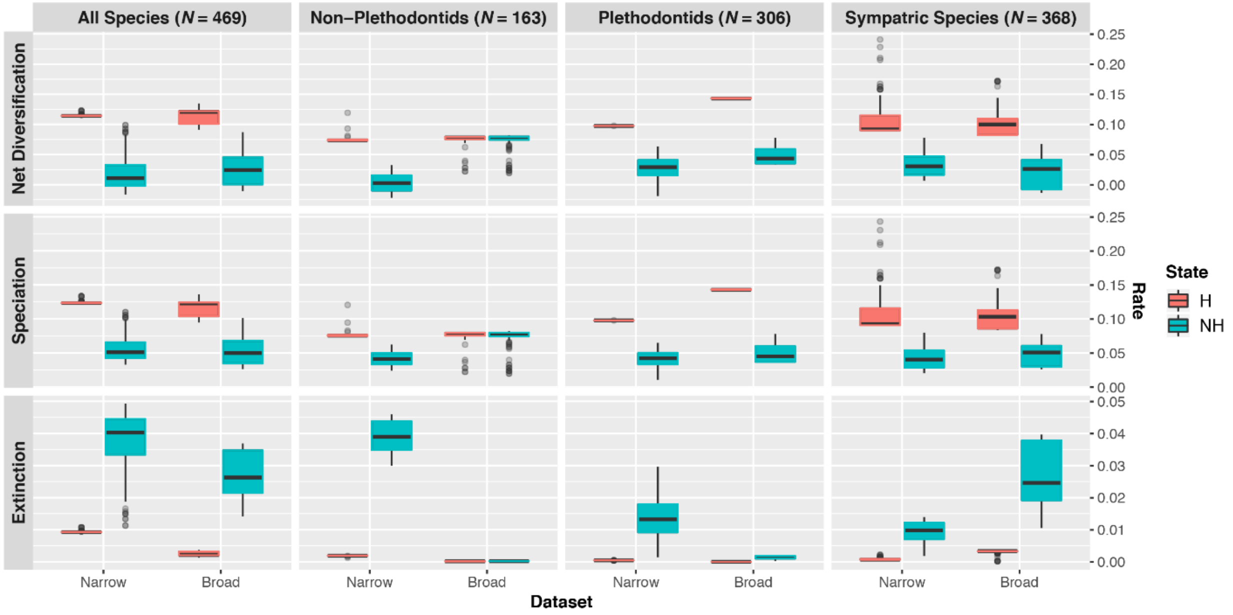

Figure S2. Model-averaged lineage-specific diversification rate estimates at the tips of the phylogeny assuming we have sampled all extant hybridizing salamanders. Results using different trees are displayed by column, whereas results for different parameters are displayed by row. Hybridizing lineages (H) are displayed in red, whereas non-hybridizing (NH) lineages are displayed in blue.

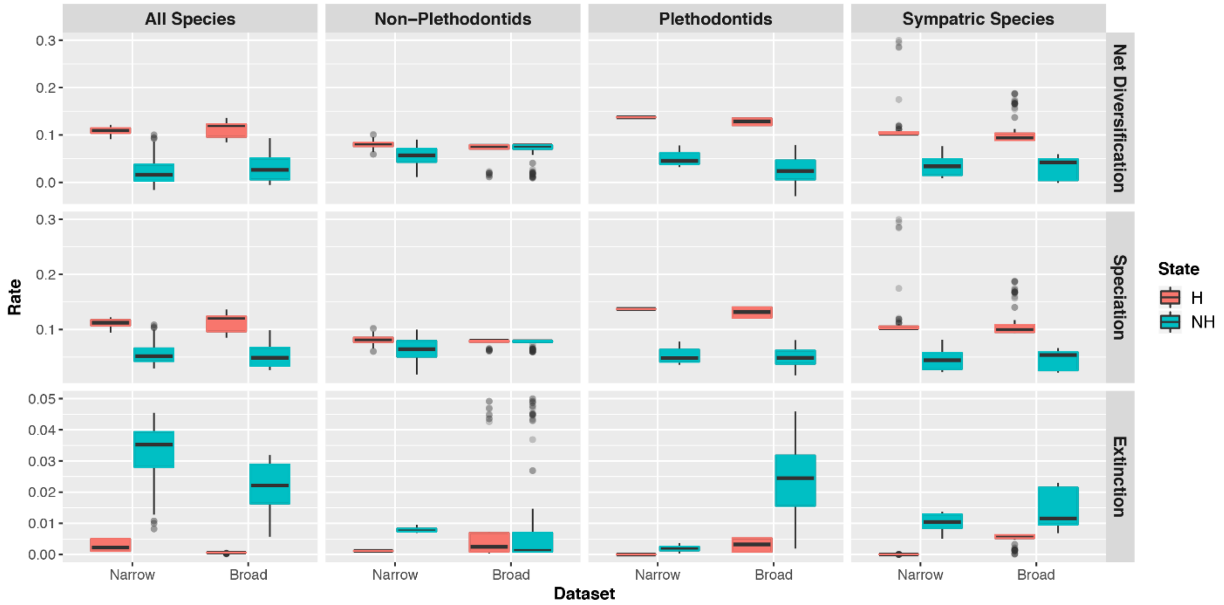

Figure S3. Model-averaged lineage-specific diversification rate estimates at the tips of the phylogeny assuming the frequency of hybridization is equal to that observed in our data. Results using different trees are displayed by column, whereas results for different parameters are displayed by row. Hybridizing lineages (H) are displayed in red, whereas non-hybridizing (NH) lineages are displayed in blue.

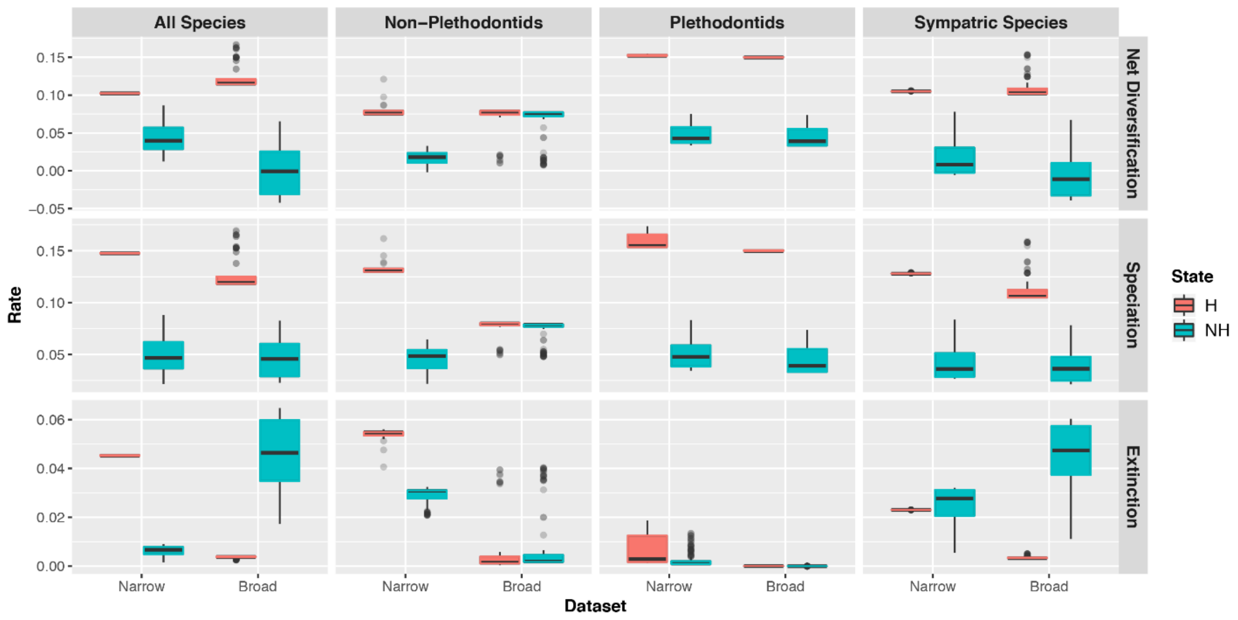

Figure S4. Model-averaged lineage-specific diversification rate estimates at the tips of the phylogeny assuming 30% of species hybridize. Results using different trees are displayed by column, whereas results for different parameters are displayed by row. Hybridizing lineages (H) are displayed in red, whereas non-hybridizing (NH) lineages are displayed in blue.

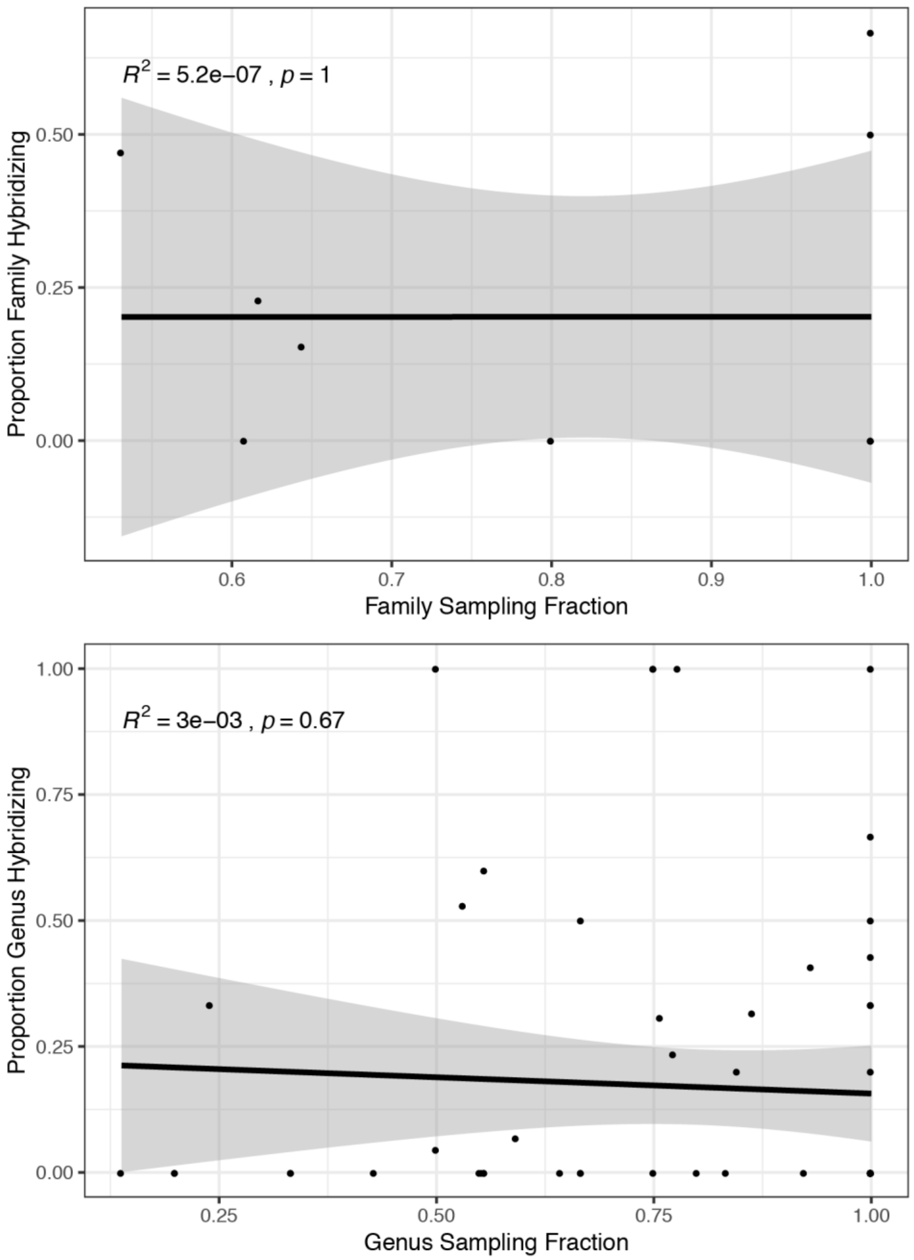

Figure S5. Relationship between clade-specific sampling fraction and prevalence of hybridization. The top plot shows results at the family level, whereas the bottom plot shows results at the genus level. No relationship is found for either dataset. Sampling fraction was defined as the proportion of extant diversity for each family/genus sampled in our phylogeny. Proportion family/genus hybridizing was defined as the proportion of extant family/genus diversity observed to hybridize in our dataset.

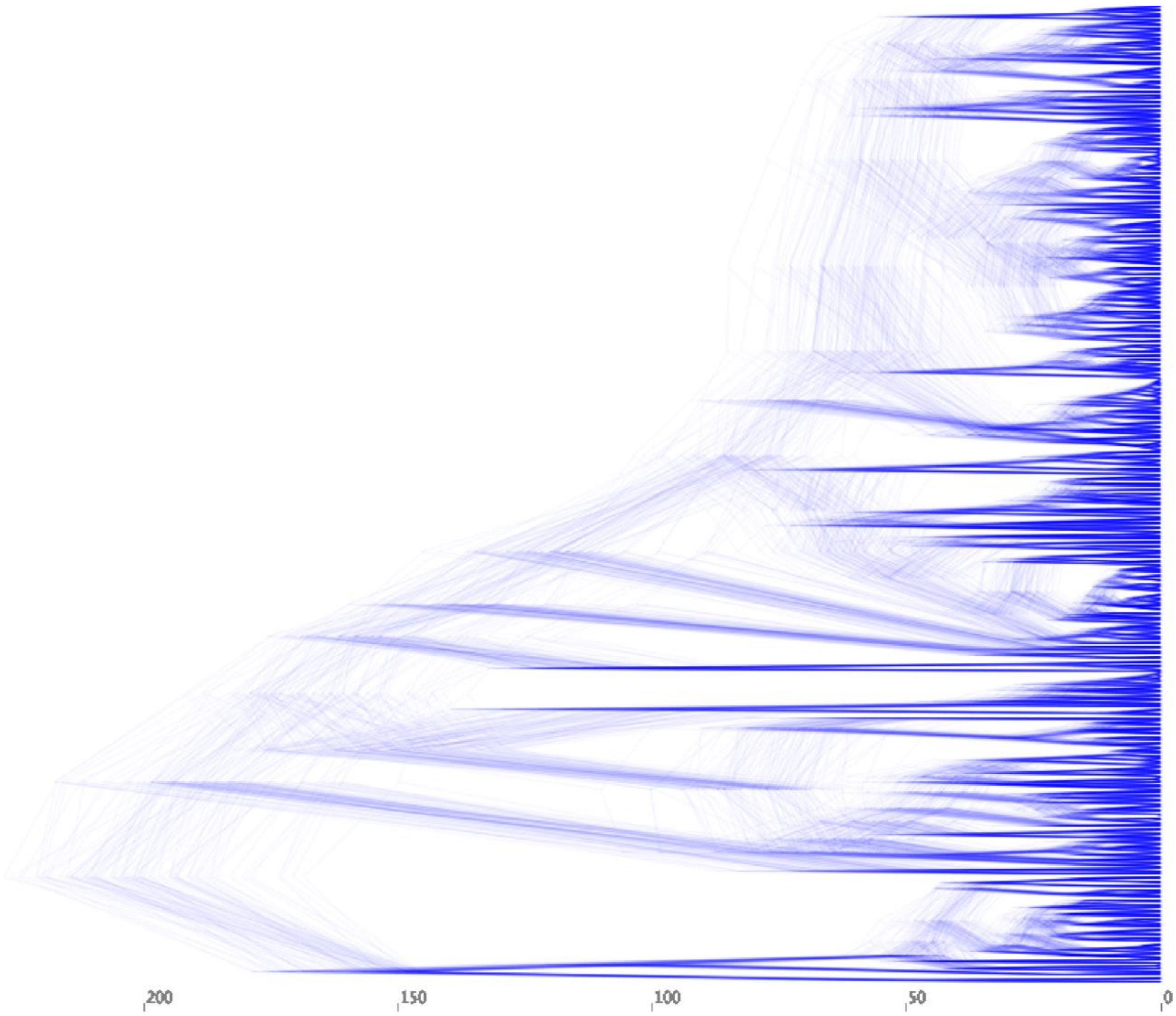

Figure S6. Posterior distribution of 100 trees used to assess phylogenetic uncertainty. Trees are obtained from Jetz and Pyron (2018).

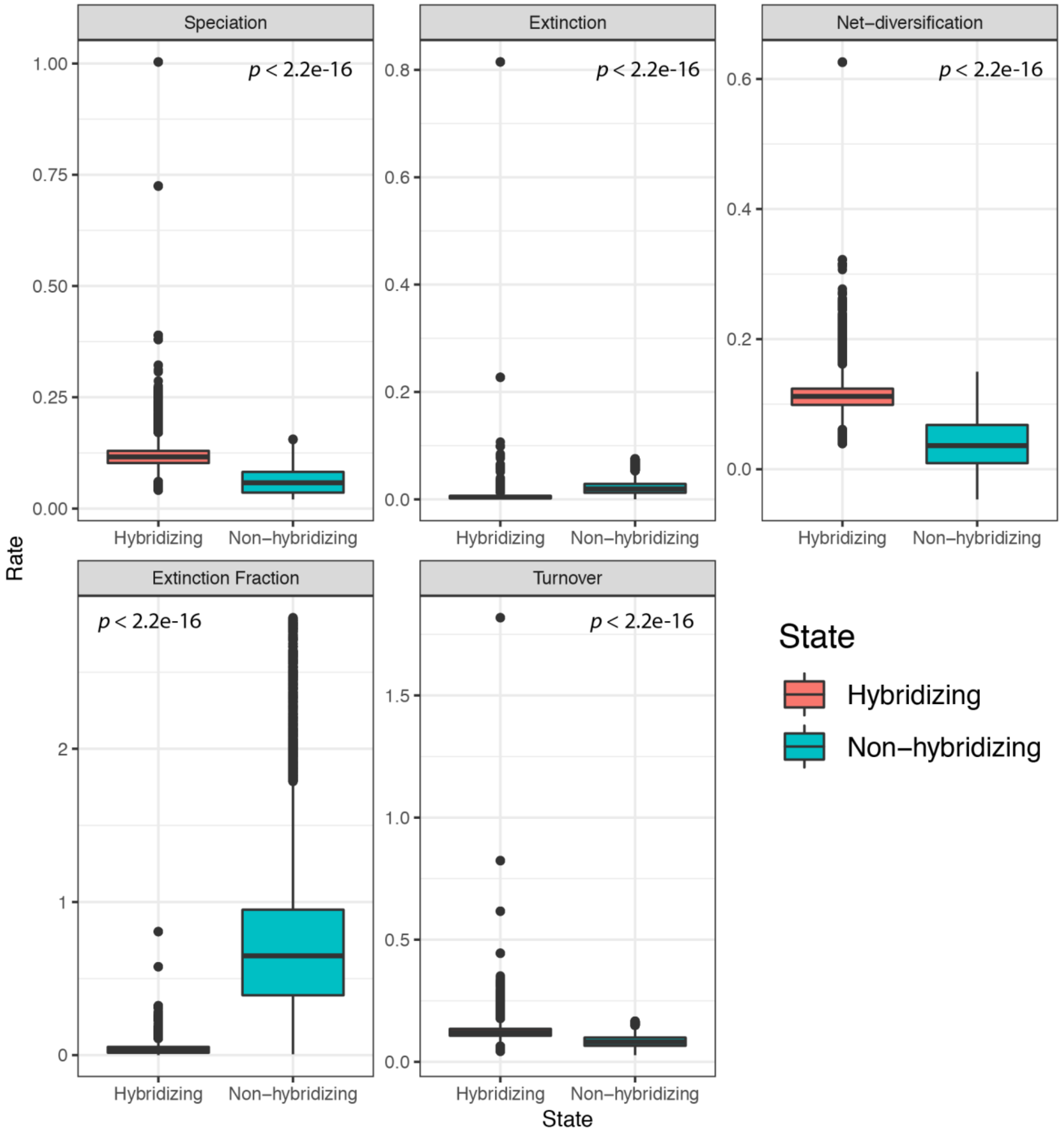

Figure S7. Diversification rates obtained by analysis of the 100 trees obtained from Jetz and Pyron (2018). Hybridizing lineages (H) are displayed in red, whereas non-hybridizing (NH) lineages are displayed in blue. P-values correspond to those obtained using a Welch's two-sample t-test using pooled model-averaged diversification rates across replicates.

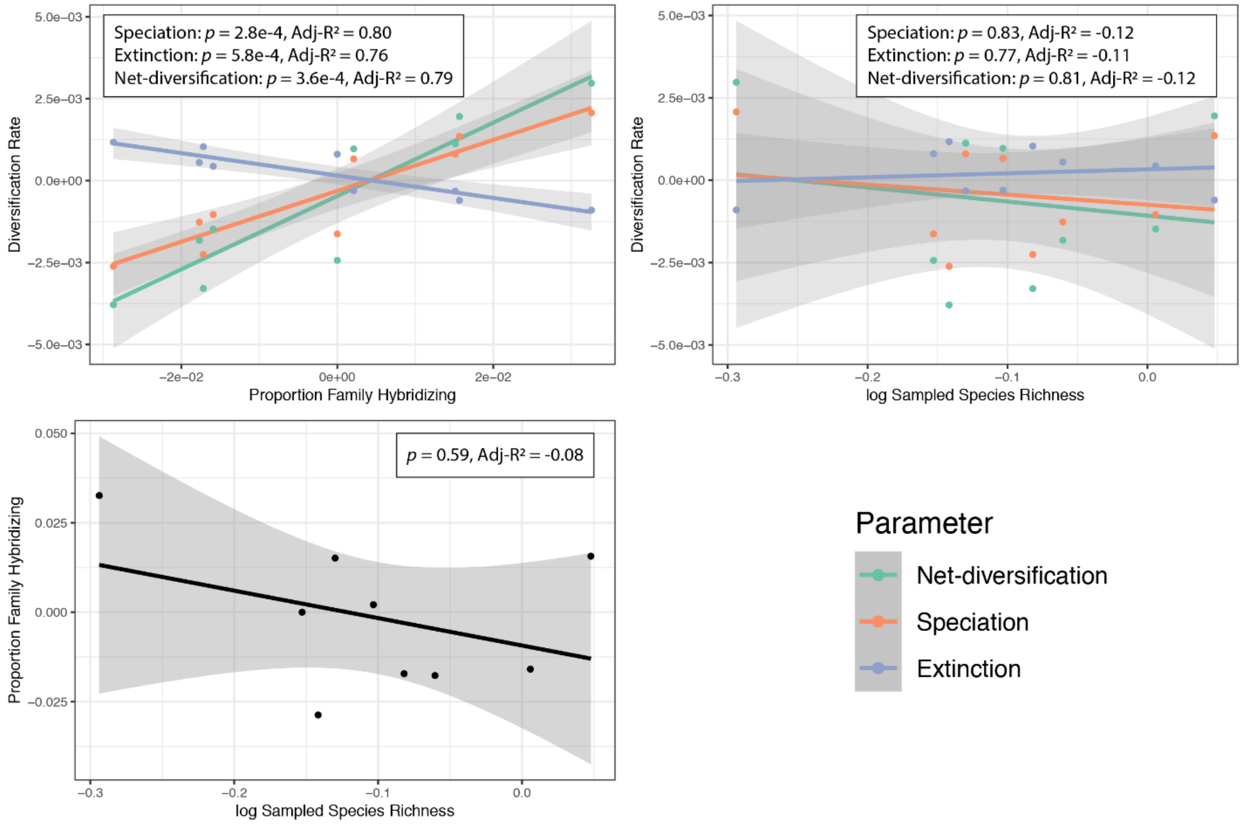

Figure S8. Relationships between A) family-level diversification rate and proportion family hybridizing, B) family-level diversification rate and species richness, and C) proportion of family hybridizing and species richness. Regressions and points are colored by rate (see inset legends).

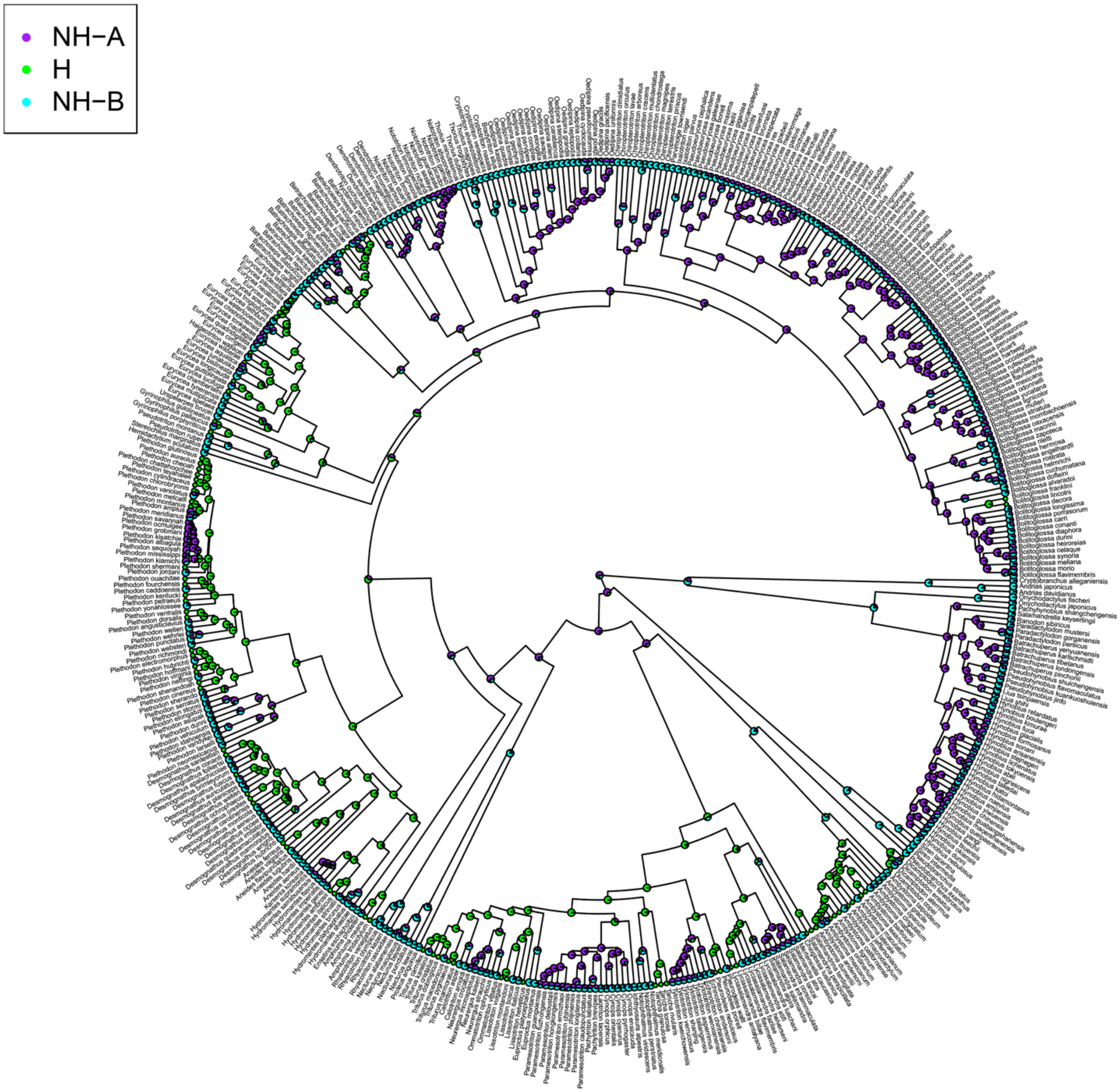

Figure S9. Marginal ancestral reconstruction of character states according to the best-fit model as implemented in HiSSE using the dataset including all taxa. Tip and node states represent their respective probabilities of belonging to each of the four states included in the full HiSSE model. The two hidden states are indicated by (**A**) and (**B**) respectively.

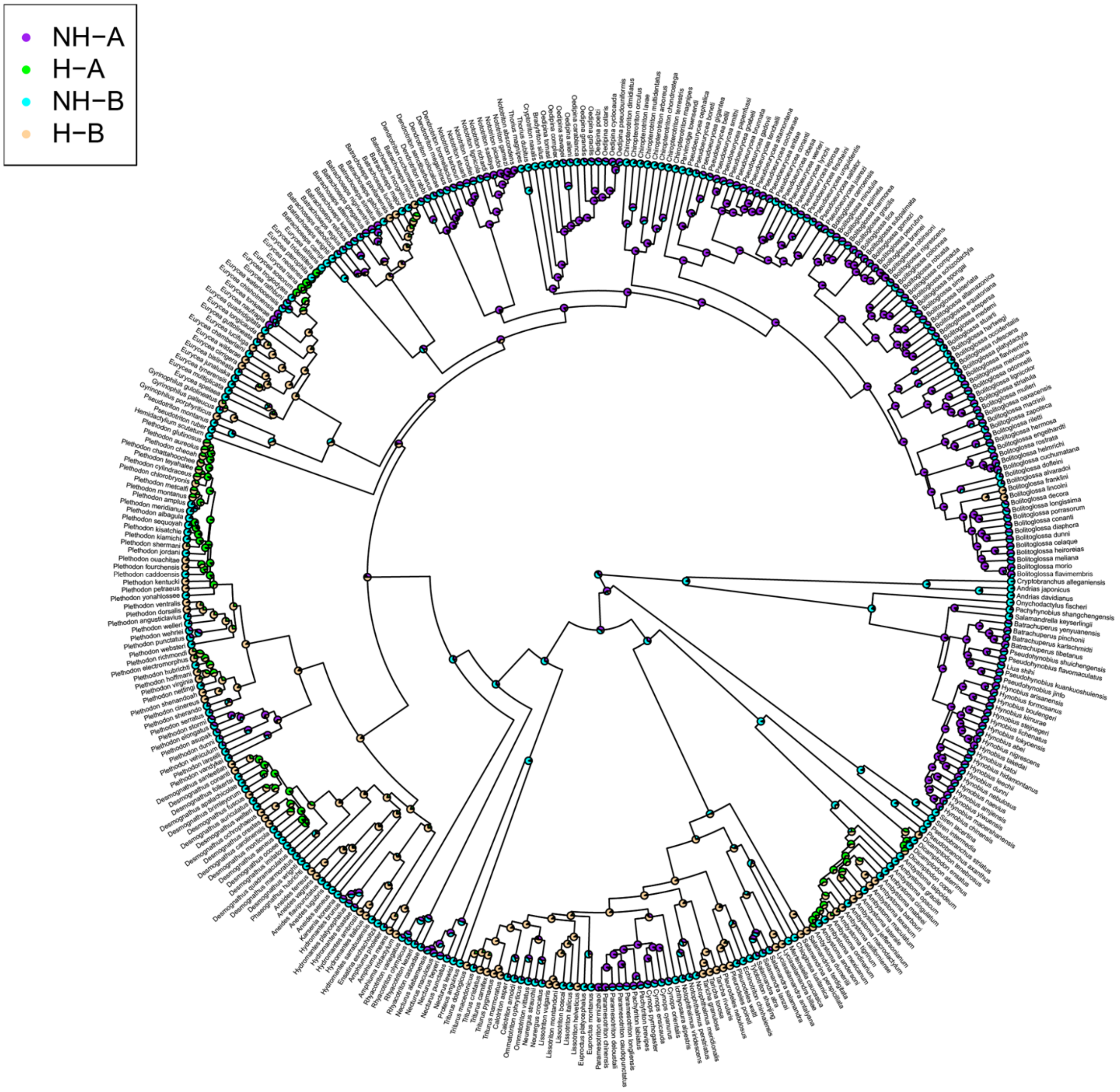

Figure S10. Marginal ancestral reconstruction of character states according to the best-fit model as implemented in HiSSE using the dataset including only sympatric taxa. Tip and node states represent their respective probabilities of belonging to each of the four states included in the full HiSSE model. The two hidden states are indicated by (**A**) and (**B**) respectively.

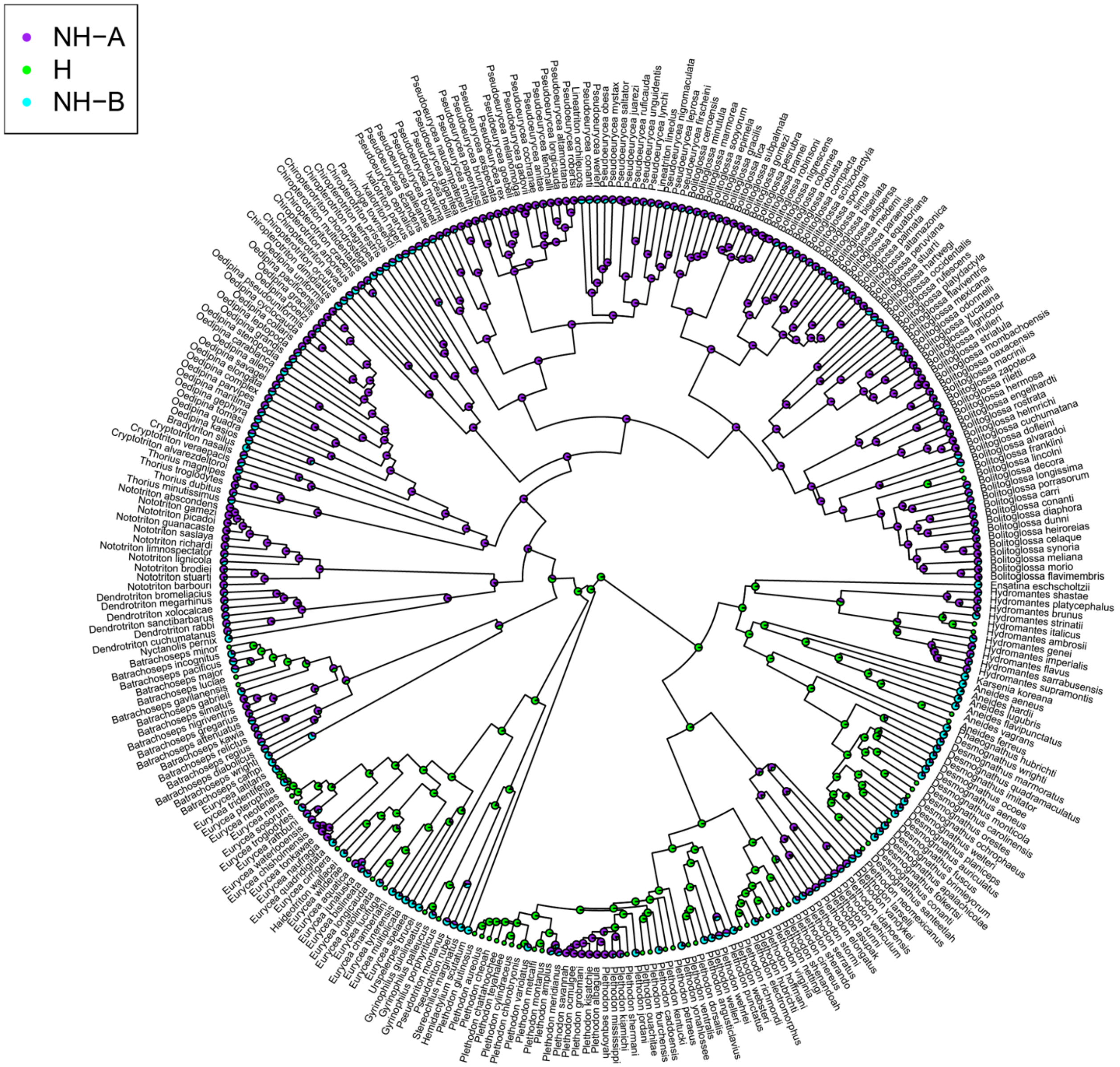

Figure S11. Marginal ancestral reconstruction of character states according to the best-fit model as implemented in HiSSE using the dataset including only plethodontids. Tip and node states represent their respective probabilities of belonging to each of the four states included in the full HiSSE model. The two hidden states are indicated by (**A**) and (**B**) respectively.

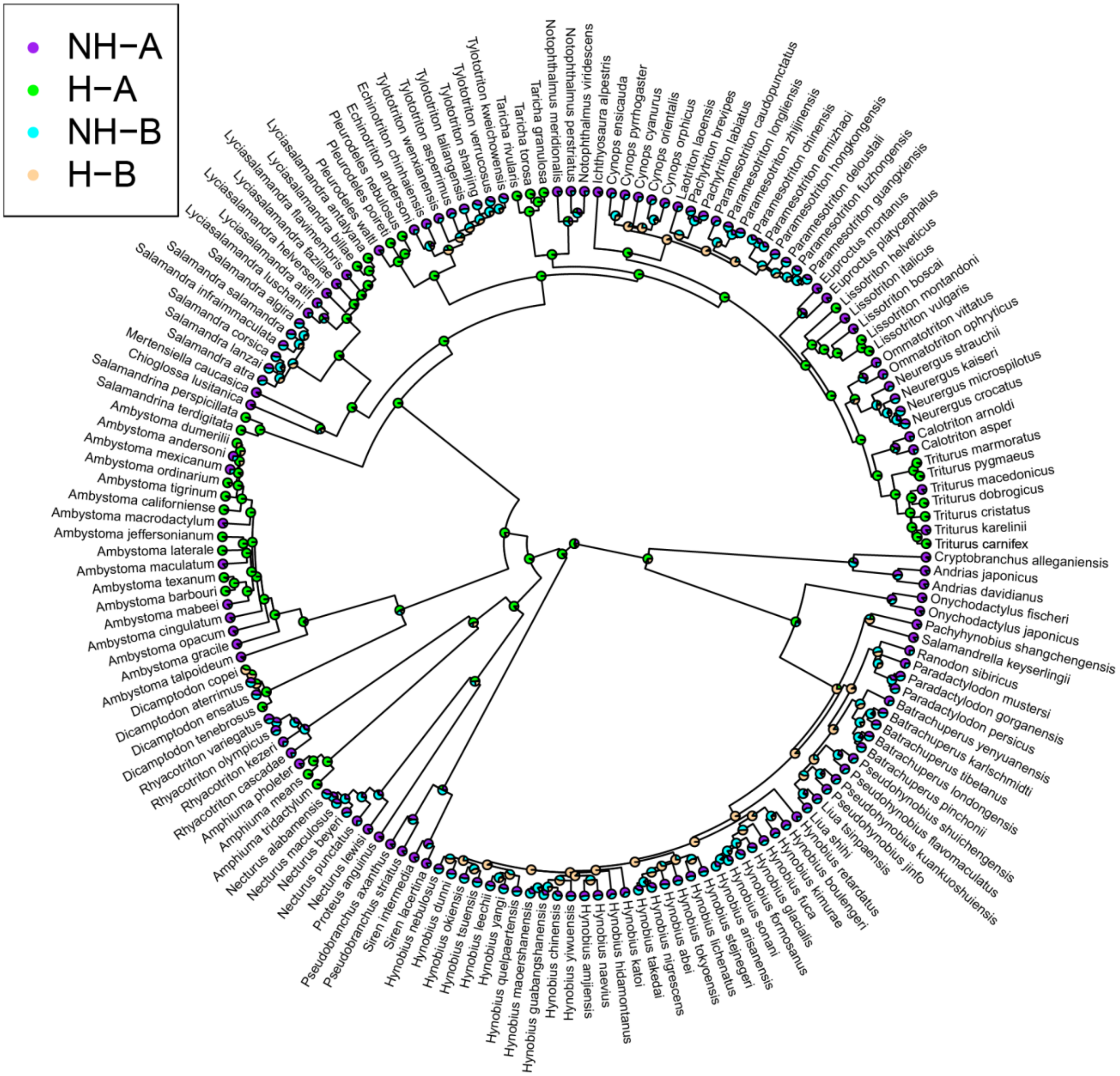

Figure S12. Marginal ancestral reconstruction of character states according to the best-fit model as implemented in HiSSE using the dataset excluding plethodontids. Tip and node states represent their respective probabilities of belonging to each of the four states included in the full HiSSE model. The two hidden states are indicated by (**A**) and (**B**) respectively.

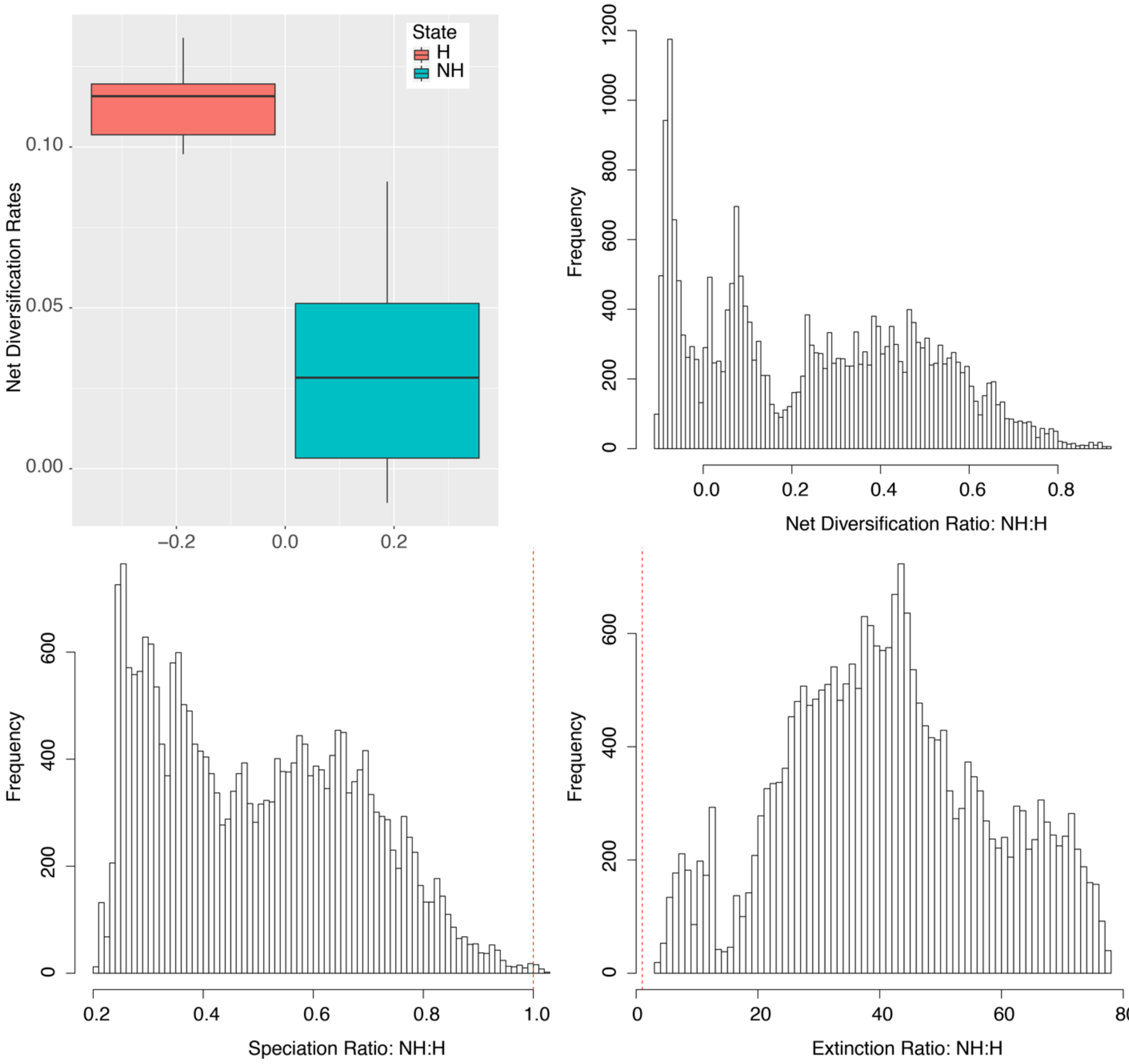

Figure S13. Comparison of model averaged parameter estimates among character states excluding the *Glutinosus* group within genus *Plethodon* and using the narrow definition of hybridization. Results reported here are those obtained when, assuming 20% of species hybridize. The box plots in the top left show model averaged net diversification rates for each state at the tips of the tree. The histograms illustrate the distributions of non-hybridizing to hybridizing lineages diversification rates as estimated at the tips of the phylogeny. A value > 1 corresponds to a comparison in which non-hybridizing lineages experience rates greater than those of hybridizing lineages and vice-versa. Dotted vertical lines are placed at 1, at which rates are equal among states.

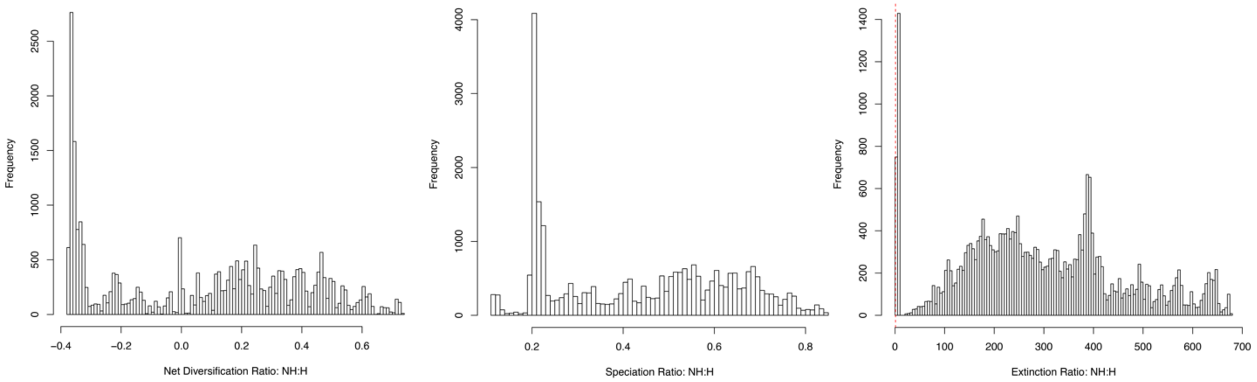

Figure S13. Comparison of model averaged parameter estimates among character states excluding the *Glutinosus* group within genus *Plethodon* and using the broad definition of hybridization. Results reported here are those obtained when, assuming 20% of species hybridize. Histograms illustrate the distributions of non-hybridizing to hybridizing lineages diversification rates as estimated at the tips of the phylogeny. A value > 1 corresponds to a comparison in which non-hybridizing lineages experience rates greater than those of hybridizing lineages and vice-versa. Dotted vertical lines are placed at 1, at which rates are equal among states.

**Cryptobranchidae**	**Narrow**	**Broad**	**Reference**
Andrias davidianus	0	1	Fukumoto et al. 2015
Andrias japonicus	0	1	Fukumoto et al. 2015
**Hynobiidae**			
Salamandrella keyserlingii	1	1	Malyarchuk et al. 2015
Hynobius tokyoensis	0	1	Kawamura & Toshijiro 1957
Hynobius nigrescens	0	1	Kawamura & Toshijiro 1957
**Dicamptodontidae**			
Dicamptodon tenebrosus	1	1	Steele et al. 2008
Dicamptodon copei	1	1	Steele et al. 2008
**Ambystomatidae**			
Ambystoma jeffersonianum	1	1	Bogart et al. 2007
Ambystoma laterale	1	1	Bogart et al. 2007
Ambystoma texanum	1	1	Eastman et al. 2009
Ambystoma barbouri	1	1	Bogart et al. 2007; Eastman et al. 2009
Ambystoma californiense	1	1	Fitzpatrick and Shaffer 2007
Ambystoma tigrinum	1	1	Bogart et al. 2007
Ambystoma ordinarium	1	1	Weisrock et al. 2006
Ambystoma mexicanum	0	1	Brandon 1972
Ambystoma dumerilii	1	1	Weisrock et al. 2006
**Salamandridae**			
Salamandrina perspicillata	1	1	Canestrelli et al. 2014; Hauswaldt et al. 2011
Salamandrina terdigitata	1	1	Canestrelli et al. 2014; Hauswaldt et al. 2011
Lyciasalamandra helverseni	0	1	Veith et al. 2008
Lyciasalamandra antalyana	1	1	Johannesen et al. 2006
Lyciasalamandra billae	1	1	Johannesen et al. 2006
Pleurodeles nebulosus	1	1	Escoriza et al. 2016
Pleurodeles poireti	1	1	Escoriza et al. 2016
Tylototriton shanjing	0	1	Phimmachak et al. 2015
Tylototriton verrucosus	0	1	Phimmachak et al. 2015
Taricha rivularis	1	1	Davis and Twitty 1964; Hedgecock and Ayala 1974; Kuchta and Tan 2005, 2006; Kuchta 2007
Taricha granulosa	1	1	Davis and Twitty 1964; Hedgecock and Ayala 1974; Kuchta and Tan 2005, 2006; Kuchta 2007
Taricha torosa	1	1	Davis and Twitty 1964; Hedgecock and Ayala 1974; Kuchta and Tan 2005, 2006; Kuchta 2007
Lissotriton helveticus	1	1	Johanet et al. 2011
Lissotriton vulgaris	1	1	Babik et al. 2005; Steinfartz et al. 2007
Lissotriton montandoni	1	1	Babik et al. 2005; Steinfartz et al. 2007
Ommatotriton ophryticus	0	1	van Riemsdijk et al. 2017
Triturus pygmaeus	1	1	Themudo et al. 2012
Triturus marmoratus	1	1	Schoorl and Zuiderwijk 1981; Brede et al. 2000; Babik et al. 2005
Triturus dobrogicus	1	1	Schoorl and Zuiderwijk 1981; Brede et al. 2000; Babik et al. 2005
Triturus macedonicus	0	1	Wielstra and Arntzen 2012
Triturus cristatus	1	1	Schoorl and Zuiderwijk 1981; Brede et al. 2000; Babik et al. 2005
Triturus carnifex	1	1	Schoorl and Zuiderwijk 1981; Brede et al. 2000; Babik et al. 2005
Triturus karelinii	0	1	Wielstra and Arntzen 2012
**Proteidae**			
Necturus maculosus	0	1	Nelson et al. 2017; Hybridization between N. maculosus and a new form (not yet described species) that is related to/sister to N. lewisi and N.punctatus.
**Amphiumidae**			
Amphiuma tridactylum	1	1	Bonett et al. 2009
Amphiuma means	1	1	Bonett et al. 2009
**Plethodontidae**			
Hydromantes ambrosii	1	1	Nascetti et al. 1996; Tilley 1998
Hydromantes italicus	1	1	Nascetti et al. 1996; Tilley 1998
Aneides ferreus	1	1	Jackman 1998
Aneides vagrans	1	1	Jackman 1998
Desmognathus ochrophaeus	1	1	Kozak 2003; Bonett 2002
Desmognathus orestes	1	1	Kozak 2003; Bonett 2002
Desmognathus carolinensis	1	1	Kozak 2003; Bonett 2002
Desmognathus fuscus	1	1	Kozak 2003; Bonett 2002
Desmognathus santeetlah	1	1	Kozak 2003; Bonett 2002
Desmognathus conanti	1	1	Kozak 2003; Bonett 2002
Plethodon shenandoah	1	1	Highton 2012
Plethodon cinereus	1	1	Highton 1999
Plethodon hoffmani	1	1	Highton 1999
Plethodon virginia	1	1	Highton 1999
Plethodon richmondi	1	1	Highton 1999
Plethodon electromorphus	1	1	Highton 1999
Plethodon ventralis	1	1	Highton 1997
Plethodon dorsalis	1	1	Highton 1997
Plethodon yonahlossee	1	1	Highton 1995; Highton and Peabody 2000; Wiens et al. 2006
Plethodon petraeus	1	1	Highton 1995; Highton and Peabody 2000; Wiens et al. 2006
Plethodon kentucki	1	1	Highton 1995; Highton and Peabody 2000; Wiens et al. 2006
Plethodon ouachitae	1	1	Shepard et al. 2011
Plethodon fourchensis	1	1	Shepard et al. 2011
Plethodon jordani	1	1	Highton 1995; Highton and Peabody 2000; Wiens et al. 2006
Plethodon shermani	1	1	Highton 1995; Highton and Peabody 2000; Wiens et al. 2006
Plethodon metcalfi	1	1	Highton 1995; Highton and Peabody 2000; Wiens et al. 2006
Plethodon montanus	1	1	Highton 1995; Highton and Peabody 2000; Wiens et al. 2006
Plethodon chlorobryonis	1	1	Highton 1995; Highton and Peabody 2000; Wiens et al. 2006
Plethodon teyahalee	1	1	Highton 1995; Highton and Peabody 2000; Wiens et al. 2006
Plethodon cylindraceus	1	1	Highton 1995; Highton and Peabody 2000; Wiens et al. 2006
Plethodon chattahoochee	1	1	Highton 1995; Highton and Peabody 2000; Wiens et al. 2006
Plethodon cheoah	1	1	Highton 1995; Highton and Peabody 2000; Wiens et al. 2006
Plethodon glutinosus	1	1	Highton 1995; Highton and Peabody 2000; Wiens et al. 2006
Plethodon aureolus	1	1	Highton et al. 2012
Gyrinophilus porphyriticus	0	1	Niemiller et al. 2008; Kuchta et al 2016
Gyrinophilus gulolineatus	0	1	Niemiller et al. 2008; Kuchta et al 2016
Gyrinophilus palleucus	0	1	Niemiller et al. 2008
Eurycea lucifuga	1	1	Smith 1964
Eurycea longicauda	1	1	Smith 1964
Eurycea wilderae	1	1	Sweet 1984; Guttman and Karlin 1986; Kozak and Montanucci 2001
Eurycea cirrigera	1	1	Sweet 1984; Guttman and Karlin 1986; Kozak and Montanucci 2001
Eurycea sosorum	1	1	Bendik et al. 2013
Eurycea nana	1	1	Bendik et al. 2013
Eurycea neotenes	1	1	Sweet 1984; Guttman and Karlin 1986; Kozak and Montanucci 2001
Eurycea tridentifera	1	1	Sweet 1984; Guttman and Karlin 1986; Kozak and Montanucci 2001
Batrachoseps simatus	1	1	Jockusch et al. 2012
Batrachoseps gavilanensis	1	1	Jockusch and Wake 2002
Batrachoseps luciae	1	1	Jockusch et al. 2012; Jockusch et al., 2001
Batrachoseps minor	1	1	Jockusch et al. 2001
Bolitoglossa lincolni	1	1	Wake and Lynch 1982; Wake et al. 1980
Bolitoglossa franklini	1	1	Wake and Lynch 1982; Wake et al. 1980

Table S1: Species designation as hybridizable according to the narrow and broad datasets and corresponding literature. Note this includes only those species included in our phylogeny

Model	Trait-dependent Diversification	All Species			Sympatric Species			Plethodontids			Non-Plethodontids		
		AICc	*ΔAICc*	*Akaike Weights*	*AICc*	*ΔAICc*	*Akaike Weights*	*AICc*	*ΔAICc*	*Akaike Weights*	*AICc*	*ΔAICc*	*Akaike Weights*
BiSSE Null: One transition	No	4173.37	182.74	0.000	3391.76	144.62	0.000	2635.67	83.12	0.000	1491.98	56.75	0.000
BiSSE Null: Two transitions	No	4090.55	99.92	0.000	3324.28	77.14	0.000	2577.00	24.46	0.000	1467.11	31.89	0.000
CID-2: All transitions, all equal	No	4117.81	127.17	0.000	3353.04	105.90	0.000	2630.00	77.45	0.000	1466.64	31.42	0.000
CID-2: Three transitions, no double	No	4021.40	30.77	0.000	3273.42	26.28	0.000	2562.99	10.44	0.004	1447.67	12.44	0.002
CID-2: No double transitions, all equal	No	4104.69	114.05	0.000	3340.20	93.06	0.000	2628.93	76.39	0.000	1462.82	27.59	0.000
CID-4: Three transitions	No	4011.10	20.47	0.000	3269.79	22.65	0.000	2560.74	8.19	0.013	1443.78	8.55	0.012
CID-4: Equal transitions	No	4087.30	96.67	0.000	3329.69	82.55	0.000	2618.14	65.59	0.000	1461.35	26.13	0.000
BiSSE: All parameters free	Yes	4061.35	70.72	0.000	3287.58	40.44	0.000	2558.34	5.80	0.044	1459.86	24.64	0.000
HiSSE: No NH-B, all transitions	Yes	4073.86	83.22	0.000	3300.24	53.10	0.000	2568.95	16.40	0.000	1493.21	57.99	0.000
HiSSE: No NH-B, no double transitions	Yes	4033.85	43.22	0.000	3277.22	30.08	0.000	2560.89	8.35	0.012	1463.94	28.71	0.000
HiSSE: No H-B, all transitions	Yes	3992.96	2.33	0.126	3292.19	45.05	0.000	2556.66	4.11	0.102	1454.11	18.88	0.000
HiSSE: No H-B, no double transitions	Yes	**3990.63**	**0.00**	**0.402**	**3247.14**	**0.00**	**0.556**	**2552.55**	**0.00**	**0.793**	**1435.22**	**0.00**	**0.879**
HiSSE: All parameters free	Yes	3994.05	3.42	0.073	3247.66	0.52	0.429	2563.94	11.40	0.003	1448.10	12.88	0.001
HiSSE: No double transitions	Yes	3990.65	0.01	0.399	3254.43	7.29	0.015	2559.15	6.60	0.029	1439.46	4.24	0.106

Table S2: The 14 models fitted in our analysis using the narrow dataset assuming we have sampled all species that hybridize in nature. Models varied in number of hidden states included as well as in the number of unique transitions among states permitted. HiSSE models contain hidden states, whereas BiSSE models do not. Double transitions are those in which two-state jumps occur (i.e. NH-**A** to H-B or vice versa). CID stands for character independent, thus CID-2 and CID-4 represent the two novel null models of trait-independent diversification that may be implemented in the HiSSE framework. Rows in bold indicate best-fit models.

Model	Trait-dependent Diversification	All Species			Sympatric Species			Plethodontids			Non-Plethodontids		
		AICc	*ΔAICc*	*Akaike Weights*	*AICc*	*ΔAICc*	*Akaike Weights*	*AICc*	*ΔAICc*	*Akaike Weights*	*AICc*	*ΔAICc*	*Akaike Weights*
BiSSE Null: One transition	No	4210.44	154.20	0.000	3443.09	122.04	0.000	2646.49	77.97	0.000	1515.40	35.86	0.000
BiSSE Null: Two transitions	No	4148.12	91.88	0.000	3393.87	72.82	0.000	2592.99	24.47	0.000	1502.54	23.00	0.000
CID-2: All transitions, all equal	No	4151.24	95.00	0.000	3401.87	80.82	0.000	2633.68	65.16	0.000	1489.38	9.84	0.005
CID-2: Three transitions, no double	No	4077.94	21.70	0.000	3342.23	21.18	0.000	2579.14	10.61	0.003	1483.82	4.27	0.081
CID-2: No double transitions, all equal	No	4139.34	83.10	0.000	3389.73	68.68	0.000	2639.53	71.01	0.000	1487.11	7.57	0.016
CID-4: Three transitions	No	4069.63	13.39	0.001	3341.48	20.43	0.000	2577.48	8.95	0.007	**1479.54**	**0.00**	**0.683**
CID-4: Equal transitions	No	4125.26	69.02	0.000	3381.94	60.89	0.000	2629.63	61.11	0.000	1489.02	9.47	0.006
BiSSE: All parameters free	Yes	4119.74	63.50	0.000	3357.57	36.52	0.000	2574.13	5.60	0.040	1510.44	30.90	0.000
HiSSE: No NH-B, all transitions	Yes	4132.25	76.01	0.000	3366.68	45.63	0.000	2580.13	11.60	0.002	1516.55	37.01	0.000
HiSSE: No NH-B, no double transitions	Yes	4089.76	33.52	0.000	3347.88	26.83	0.000	2576.99	8.47	0.010	1506.97	27.43	0.000
HiSSE: No H-B, all transitions	Yes	4058.84	2.60	0.144	3368.30	47.25	0.000	**2568.52**	**0.00**	**0.658**	1494.62	15.08	0.000
HiSSE: No H-B, no double transitions	Yes	4057.57	1.33	0.273	3325.01	3.96	0.087	2570.40	1.88	0.257	1482.56	3.02	0.151
HiSSE: All parameters free	Yes	**4056.24**	**0.00**	**0.530**	**3321.05**	**0.00**	**0.630**	2581.88	13.36	0.001	1489.90	10.36	0.004
HiSSE: No double transitions	Yes	4060.85	4.62	0.053	3322.65	1.60	0.283	2575.38	6.85	0.021	1484.58	5.03	0.055

Table S3: The 14 models fitted in our analysis using the narrow dataset assuming we have sampled all species that hybridize in nature. Models varied in number of hidden states included as well as in the number of unique transitions among states permitted. HiSSE models contain hidden states, whereas BiSSE models do not. Double transitions are those in which two-state jumps occur (i.e. NH-**A** to H-B or vice versa). CID stands for character independent, thus CID-2 and CID-4 represent the two novel null models of trait-independent diversification that may be implemented in the HiSSE framework. Rows in bold indicate best-fit models.

Model	Trait-dependent Diversification	All Species			Sympatric Species			Plethodontids			Non-Plethodontids		
		AICc	*ΔAICc*	*Akaike Weights*	*AICc*	*ΔAICc*	*Akaike Weights*	*AICc*	*ΔAICc*	*Akaike Weights*	*AICc*	*ΔAICc*	*Akaike Weights*
BiSSE Null: One transition	No	4169.69	181.78	0.000	3390.54	153.16	0.000	2637.46	94.86	0.000	1486.77	49.77	0.000
BiSSE Null: Two transitions	No	4093.52	105.61	0.000	3329.65	92.27	0.000	2582.39	39.79	0.000	1464.96	27.96	0.000
CID-2: All transitions, all equal	No	4112.63	124.72	0.000	3350.40	113.02	0.000	2623.60	81.00	0.000	1461.89	24.89	0.000
CID-2: Three transitions, no double	No	4023.51	35.60	0.000	3278.34	40.97	0.000	2565.84	23.24	0.000	1437.56	0.56	0.383
CID-2: No double transitions, all equal	No	4098.26	110.35	0.000	3336.68	99.30	0.000	2627.33	84.73	0.000	1458.20	21.20	0.000
CID-4: Three transitions	No	4018.18	30.27	0.000	3275.63	38.25	0.000	2567.99	25.39	0.000	1444.34	7.34	0.013
CID-4: Equal transitions	No	4093.95	106.04	0.000	3334.87	97.49	0.000	2624.46	81.86	0.000	1464.29	27.29	0.000
BiSSE: All parameters free	Yes	4047.43	59.52	0.000	3272.86	35.49	0.000	2552.10	9.50	0.008	1457.53	20.53	0.000
HiSSE: No NH-B, all transitions	Yes	4040.95	53.04	0.000	3284.37	47.00	0.000	2564.89	22.29	0.000	1471.08	34.07	0.000
HiSSE: No NH-B, no double transitions	Yes	4023.47	35.55	0.000	3268.31	30.94	0.000	2559.83	17.23	0.000	1446.69	9.69	0.004
HiSSE: No H-B, all transitions	Yes	4059.91	71.99	0.000	3246.77	9.39	0.008	2546.72	4.12	0.112	1440.89	3.88	0.073
HiSSE: No H-B, no double transitions	Yes	3988.53	0.62	0.380	3251.58	14.21	0.001	**2542.60**	**0.00**	**0.875**	1443.75	6.75	0.017
HiSSE: All parameters free	Yes	**3987.91**	**0.00**	**0.518**	3241.24	3.86	0.125	2556.65	14.05	0.001	1446.79	9.78	0.004
HiSSE: No double transitions	Yes	3991.16	3.24	0.102	**3237.38**	**0.00**	**0.866**	2552.92	10.32	0.005	**1437.00**	**0.00**	**0.506**

Table S4: The 14 models fitted in our analysis using the narrow dataset assuming the prevalence of hybridization in nature is equal to that which we observe in our data. Models varied in number of hidden states included as well as in the number of unique transitions among states permitted. HiSSE models contain hidden states, whereas BiSSE models do not. Double transitions are those in which two-state jumps occur (i.e. NH-**A** to H-B or vice versa). CID stands for character independent, thus CID-2 and CID-4 represent the two novel null models of trait-independent diversification that may be implemented in the HiSSE framework. Rows in bold indicate best-fit models.

Model	Trait-dependent Diversification	All Species			Sympatric Species			Plethodontids			Non-Plethodontids		
		AICc	*ΔAICc*	*Akaike Weights*	*AICc*	*ΔAICc*	*Akaike Weights*	*AICc*	*ΔAICc*	*Akaike Weights*	*AICc*	*ΔAICc*	*Akaike Weights*
BiSSE Null: One transition	No	4209.37	162.16	0.000	3443.21	132.92	0.000	2649.83	99.41	0.000	1510.60	36.34	0.000
BiSSE Null: Two transitions	No	4152.32	105.11	0.000	3400.60	90.31	0.000	2599.40	48.98	0.000	1500.97	26.72	0.000
CID-2: All transitions, all equal	No	4149.73	102.52	0.000	3401.32	91.03	0.000	2641.06	90.65	0.000	1488.27	14.02	0.001
CID-2: Three transitions, no double	No	4081.99	34.78	0.000	3348.52	38.22	0.000	2582.60	32.19	0.000	**1474.25**	**0.00**	**0.932**
CID-2: No double transitions, all equal	No	4137.14	89.93	0.000	3389.12	78.82	0.000	2639.06	88.65	0.000	1485.78	11.53	0.003
CID-4: Three transitions	No	4076.88	29.67	0.000	3347.10	36.81	0.000	2585.01	34.59	0.000	1480.35	6.10	0.044
CID-4: Equal transitions	No	4132.20	84.99	0.000	3387.42	77.13	0.000	2636.50	86.08	0.000	1490.92	16.66	0.000
BiSSE: All parameters free	Yes	4098.88	51.67	0.000	3340.46	30.16	0.000	2567.40	16.98	0.000	1493.79	19.54	0.000
HiSSE: No NH-B, all transitions	Yes	4110.92	63.71	0.000	3353.08	42.79	0.000	2580.19	29.77	0.000	1512.23	37.98	0.000
HiSSE: No NH-B, no double transitions	Yes	4077.76	30.55	0.000	3336.59	26.29	0.000	2575.86	25.44	0.000	1485.53	11.28	0.003
HiSSE: No H-B, all transitions	Yes	4057.10	9.89	0.006	3333.84	23.54	0.000	2562.23	11.81	0.003	1493.27	19.02	0.000
HiSSE: No H-B, no double transitions	Yes	4054.97	7.76	0.017	3325.33	15.03	0.001	**2557.05**	**6.63**	**0.035**	1482.48	8.23	0.015
HiSSE: All parameters free	Yes	**4047.21**	**0.00**	**0.847**	3315.56	5.26	0.067	2569.97	19.55	0.000	1487.54	13.28	0.001
HiSSE: No double transitions	Yes	4050.96	3.75	0.130	**3310.29**	**0.00**	**0.932**	2550.42	0.00	0.962	1504.32	30.06	0.000

Table S5: The 14 models fitted in our analysis using the broad dataset assuming the prevalence of hybridization in nature is equal to that which we observe in our data. Models varied in number of hidden states included as well as in the number of unique transitions among states permitted. HiSSE models contain hidden states, whereas BiSSE models do not. Double transitions are those in which two-state jumps occur (i.e. NH-A to H-B or vice versa). CID stands for character independent, thus CID-2 and CID-4 represent the two novel null models of trait-independent diversification that may be implemented in the HiSSE framework. Rows in bold indicate best-fit model.

Model	Trait-dependent Diversification	All Species			Sympatric Species			Plethodontids			Non-Plethodontids		
		AICc	*ΔAICc*	*Akaike Weights*	*AICc*	*ΔAICc*	*Akaike Weights*	*AICc*	*ΔAICc*	*Akaike Weights*	*AICc*	*ΔAICc*	*Akaike Weights*
BiSSE Null: One transition	No	4170.31	190.81	0.000	3391.55	150.94	0.000	2644.86	93.89	0.000	1486.77	42.49	0.000
BiSSE Null: Two transitions	No	4098.94	119.44	0.000	3334.63	94.01	0.000	2589.79	38.82	0.000	1464.96	20.68	0.000
CID-2: All transitions, all equal	No	4110.14	130.64	0.000	3349.15	108.54	0.000	2632.11	81.14	0.000	1466.48	22.20	0.000
CID-2: Three transitions, no double	No	4027.61	48.11	0.000	3281.92	41.31	0.000	2570.98	20.01	0.000	1454.05	9.77	0.005
CID-2: No double transitions, all equal	No	4096.81	117.31	0.000	3336.35	95.74	0.000	2633.89	82.91	0.000	1464.02	19.74	0.000
CID-4: Three transitions	No	4022.21	42.71	0.000	3278.06	37.45	0.000	2574.33	23.36	0.000	1449.92	5.64	0.039
CID-4: Equal transitions	No	4097.25	117.74	0.000	3338.06	97.45	0.000	2633.18	82.20	0.000	1469.52	25.24	0.000
BiSSE: All parameters free	Yes	4039.81	60.31	0.000	3268.29	27.68	0.000	2563.16	12.19	0.001	1458.20	13.92	0.001
HiSSE: No NH-B, no double transitions	Yes	4020.58	41.08	0.000	3266.29	25.68	0.000	2568.96	17.99	0.000	1459.34	15.06	0.000
HiSSE: No NH-B, all transitions	Yes	4038.08	58.58	0.000	3279.81	39.19	0.000	2575.96	24.98	0.000	1471.11	26.83	0.000
HiSSE: No H-B , no double transitions	Yes	**3979.50**	**0.00**	**0.715**	3274.05	33.44	0.000	**2550.97**	**0.00**	**0.523**	1448.35	4.07	0.085
HiSSE: No H-B, all transitions	Yes	3990.73	11.22	0.003	3249.90	9.29	0.009	2551.17	0.19	0.475	1446.57	2.29	0.206
HiSSE: All parameters free	Yes	3986.74	7.24	0.019	**3240.61**	**0.00**	**0.980**	2565.34	14.36	0.000	1451.73	7.45	0.016
HiSSE: No double transitions	Yes	3981.50	2.00	0.263	3249.66	9.05	0.011	2565.26	14.29	0.000	**1444.28**	**0.00**	**0.648**

Table S6: The 14 models fitted in our analysis using the narrow dataset assuming 20% of species hybridize. Models varied in number of hidden states included as well as in the number of unique transitions among states permitted. HiSSE models contain hidden states, whereas BiSSE models do not. Double transitions are those in which two-state jumps occur (i.e. NH-**A** to H-B or vice versa). CID stands for character independent, thus CID-2 and CID-4 represent the two novel null models of trait-independent diversification that may be implemented in the HiSSE framework. Rows in bold indicate best-fit models.

Model	Trait-dependent Diversification	All Species			Sympatric Species			Plethodontids			Non-plethodontids		
		AICc	*ΔAICc*	*Akaike Weights*	*AICc*	*ΔAICc*	*Akaike Weights*	*AICc*	*ΔAICc*	*Akaike Weights*	*AICc*	*ΔAICc*	*Akaike Weights*
BiSSE Null: One transition	No	4209.55	162.81	0.000	3442.40	123.97	0.000	2652.92	98.52	0.000	1513.34	39.60	0.000
BiSSE Null: Two transitions	No	4152.91	106.17	0.000	3395.12	76.69	0.000	2604.52	50.12	0.000	1501.47	27.74	0.000
CID-2: All transitions, all equal	No	4149.59	102.86	0.000	3401.56	83.14	0.000	2636.08	81.68	0.000	1488.83	15.10	0.000
CID-2: Three transitions, no double	No	4082.41	35.67	0.000	3343.39	24.96	0.000	2588.50	34.10	0.000	**1473.74**	**0.00**	**0.949**
CID-2: No double transitions, all equal	No	4137.09	90.36	0.000	3388.86	70.43	0.000	2638.55	84.14	0.000	1486.23	12.49	0.002
CID-4: Three transitions	No	4075.91	29.18	0.000	3355.39	36.96	0.000	2584.89	30.48	0.000	1480.55	6.82	0.031
CID-4: Equal transitions	No	4144.60	97.86	0.000	3395.84	77.41	0.000	2640.87	86.46	0.000	1490.21	16.48	0.000
BiSSE: All parameters free	Yes	4097.73	50.99	0.000	3350.59	32.16	0.000	2565.14	10.74	0.005	1510.75	37.01	0.000
HiSSE: No NH-B, all transitions	Yes	4077.04	30.31	0.000	3343.36	24.94	0.000	2573.61	19.21	0.000	1489.37	15.63	0.000
HiSSE: No NH-B, no double transitions	Yes	4076.68	29.94	0.000	3360.11	41.69	0.000	2577.93	23.53	0.000	1516.29	42.56	0.000
HiSSE: No H-B, all transitions	Yes	4049.90	3.16	0.130	3355.30	36.87	0.000	2564.85	10.45	0.005	1492.63	18.89	0.000
HiSSE: No H-B, no double transitions	Yes	4054.96	8.22	0.010	3329.95	11.52	0.002	**2554.40**	**0.00**	**0.970**	1482.37	8.64	0.013
HiSSE: All parameters free	Yes	**4046.74**	**0.00**	**0.631**	3319.57	1.14	0.361	2566.55	12.15	0.002	1488.73	15.00	0.001
HiSSE: No double transitions	Yes	4048.76	2.02	0.229	**3318.43**	**0.00**	**0.637**	2562.33	7.93	0.018	1484.90	11.17	0.004

Table S7: The 14 models fitted in our analysis using the broad dataset assuming 20% of species hybridize. Models varied in number of hidden states included as well as in the number of unique transitions among states permitted. HiSSE models contain hidden states, whereas BiSSE models do not. Double transitions are those in which two-state jumps occur (i.e. NH-A to H-B or vice versa). CID stands for character independent, thus CID-2 and CID-4 represent the two novel null models of trait-independent diversification that may be implemented in the HiSSE framework. Rows in bold indicate best-fit model.

Model	Trait-dependent Diversification	All Species			Sympatric Species			Plethodontids			Non-Plethodontids		
		AICc	*ΔAICc*	*Akaike Weights*	*AICc*	*ΔAICc*	*Akaike Weights*	*AICc*	*ΔAICc*	*Akaike Weights*	*AICc*	*ΔAICc*	*Akaike Weights*
BiSSE Null: One transition	No	4175.19	196.80	0.000	3393.81	162.88	0.000	2648.43	108.72	0.000	1482.50	40.69	0.000
BiSSE Null: Two transitions	No	4117.89	139.50	0.000	3343.74	112.81	0.000	2604.82	65.11	0.000	1468.44	26.63	0.000
CID-2: All transitions, all equal	No	4106.77	128.38	0.000	3347.79	116.87	0.000	2634.37	94.66	0.000	1457.13	15.32	0.000
CID-2: Three transitions, no double	No	4047.25	68.86	0.000	3292.72	61.79	0.000	2580.60	40.89	0.000	1448.30	6.49	0.024
CID-2: No double transitions, all equal	No	4097.28	118.89	0.000	3337.17	106.24	0.000	2628.82	89.11	0.000	1455.57	13.76	0.001
CID-4: Three transitions	No	4006.14	27.75	0.000	3282.41	51.48	0.000	2546.08	6.37	0.038	1448.71	6.90	0.019
CID-4: Equal transitions	No	4083.10	104.71	0.000	3328.09	97.16	0.000	2606.16	66.44	0.000	1472.13	30.32	0.000
BiSSE: All parameters free	Yes	4046.68	68.29	0.000	3264.66	33.73	0.000	2549.85	10.14	0.006	1457.41	15.60	0.000
HiSSE: No NH-B, all transitions	Yes	4059.20	80.81	0.000	3276.06	45.13	0.000	2658.06	118.35	0.000	1469.41	27.60	0.000
HiSSE: No NH-B, no double transitions	Yes	4020.54	42.16	0.000	3265.99	35.06	0.000	2557.47	17.76	0.000	1459.32	17.51	0.000
HiSSE: No H-B, all transitions	Yes	**3978.39**	**0.00**	**0.862**	3242.87	11.95	0.003	2547.05	7.34	0.023	1459.10	17.29	0.000
HiSSE: No H-B, no double transitions	Yes	3982.24	3.86	0.125	**3230.93**	**0.00**	**0.991**	**2539.71**	**0.00**	**0.915**	1443.25	1.44	0.298
HiSSE: All parameters free	Yes	3986.85	8.46	0.013	3240.96	10.03	0.007	2547.83	8.12	0.016	1446.99	5.18	0.046
HiSSE: No double transitions	Yes	4000.55	22.17	0.000	3250.83	19.90	0.000	2552.34	12.63	0.002	**1441.81**	**0.00**	**0.612**

Table S8: The 14 models fitted in our analysis using the narrow dataset assuming 30% of species hybridize. Models varied in number of hidden states included as well as in the number of unique transitions among states permitted. HiSSE models contain hidden states, whereas BiSSE models do not. Double transitions are those in which two-state jumps occur (i.e. NH-**A** to H-B or vice versa). CID stands for character independent, thus CID-2 and CID-4 represent the two novel null models of trait-independent diversification that may be implemented in the HiSSE framework. Rows in bold indicate best-fit models.

Model	Trait-dependent Diversification	All Species			Sympatric Species			Plethodontids			Non-Plethodontids		
		AICc	*ΔAICc*	*Akaike Weights*	*AICc*	*ΔAICc*	*Akaike Weights*	*AICc*	*ΔAICc*	*Akaike Weights*	*AICc*	*ΔAICc*	*Akaike Weights*
BiSSE Null: One transition	No	4209.37	162.16	0.000	3443.21	132.92	0.000	2649.83	99.41	0.000	1510.60	36.34	0.000
BiSSE Null: Two transitions	No	4152.32	105.11	0.000	3400.60	90.31	0.000	2599.40	48.98	0.000	1500.97	26.72	0.000
CID-2: All transitions, all equal	No	4149.73	102.52	0.000	3401.32	91.03	0.000	2641.06	90.65	0.000	1488.27	14.02	0.001
CID-2: Three transitions, no double	No	4081.99	34.78	0.000	3348.52	38.22	0.000	2582.60	32.19	0.000	**1474.25**	**0.00**	**0.932**
CID-2: No double transitions, all equal	No	4137.14	89.93	0.000	3389.12	78.82	0.000	2639.06	88.65	0.000	1485.78	11.53	0.003
CID-4: Three transitions	No	4076.88	29.67	0.000	3347.10	36.81	0.000	2585.01	34.59	0.000	1480.35	6.10	0.044
CID-4: Equal transitions	No	4132.20	84.99	0.000	3387.42	77.13	0.000	2636.50	86.08	0.000	1490.92	16.66	0.000
BiSSE: All parameters free	Yes	4098.88	51.67	0.000	3340.46	30.16	0.000	2567.40	16.98	0.000	1493.79	19.54	0.000
HiSSE: No NH-B, all transitions	Yes	4110.92	63.71	0.000	3353.08	42.79	0.000	2580.19	29.77	0.000	1512.23	37.98	0.000
HiSSE: No NH-B, no double transitions	Yes	4077.76	30.55	0.000	3336.59	26.29	0.000	2575.86	25.44	0.000	1485.53	11.28	0.003
HiSSE: No H-B, all transitions	Yes	4057.10	9.89	0.006	3333.84	23.54	0.000	2562.23	11.81	0.003	1493.27	19.02	0.000
HiSSE: No H-B, no double transitions	Yes	4054.97	7.76	0.017	3325.33	15.03	0.001	2557.05	6.63	0.035	1482.48	8.23	0.015
HiSSE: All parameters free	Yes	**4047.21**	**0.00**	**0.847**	3315.56	5.26	0.067	2569.97	19.55	0.000	1487.54	13.28	0.001
HiSSE: No double transitions	Yes	4050.96	3.75	0.130	**3310.29**	**0.00**	**0.932**	**2550.42**	**0.00**	**0.962**	1504.32	30.06	0.000

Table S9: The 14 models fitted in our analysis using the broad dataset assuming 30% of species hybridize. Models varied in number of hidden states included as well as in the number of unique transitions among states permitted. HiSSE models contain hidden states, whereas BiSSE models do not. Double transitions are those in which two-state jumps occur (i.e. NH-A to H-B or vice versa). CID stands for character independent, thus CID-2 and CID-4 represent the two novel null models of trait-independent diversification that may be implemented in the HiSSE framework. Rows in bold indicate best-fit model.

Definition of Hybridization	Dataset	Model	Akaike Weight	Speciation				Extinction				Net Diversification			
				NH-A	*NH-B*	*H-A*	*H-B*	*NH-A*	*NH-B*	*H-A*	*H-B*	*NH-A*	*NH-B*	*H-A*	*H-B*
Narrow	**All Species**	**HiSSE: No H-B, no double transitions**	**0.402**	**0.150**	**0.026**	**0.098**	**NA**	**3.09E-10**	**2.02E-10**	**0.078**	**NA**	**0.150**	**2.58E-2**	**0.021**	**NA**
		HiSSE: No double transitions	0.399	0.112	0.029	0.093	0.112	2.49E-9	0.028	1.91E-10	2.32E-10	0.112	1.44E-3	0.093	0.112
		HiSSE: No H-B, all transitions	0.126	0.023	0.126	0.104	NA	0.015	0.037	2.15E-10	NA	0.008	8.91E-2	0.104	NA
		HiSSE: All parameters free	0.073	0.118	0.032	0.132	0.051	2.44E-10	0.034	2.72E-10	1.05E-10	0.118	-1.80E-3	0.132	0.051
	**Sympatric Species**	**HiSSE: No H-B, no double transitions**	**0.556**	**0.124**	**0.021**	**0.093**	**NA**	**2.56E-10**	**0.064**	**1.91E-10**	**NA**	**0.124**	**-4.23E-2**	**0.093**	**NA**
		HiSSE: All parameters free	0.429	0.099	0.029	0.064	0.210	2.04E-10	0.033	1.32E-10	4.42E-10	0.099	-3.50E-3	0.064	0.210
	**Plethodontids**	**HiSSE: No H-B, no double transitions**	**0.793**	**0.222**	**0.059**	**0.058**	**NA**	**4.58E-10**	**1.21E-10**	**1.19E-10**	**NA**	**0.222**	**5.88E-2**	**0.058**	**NA**
		HiSSE: No H-B, all transitions	0.102	0.001	0.067	0.112	NA	0.003	1.37E-10	2.30E-10	NA	-0.002	6.66E-2	0.112	NA
	**Non-plethodontids**	**HiSSE: No H-B, no double transitions**	**0.879**	**0.157**	**0.032**	**0.069**	**NA**	**7.43E-04**	**0.096**	**0.001**	**NA**	**0.156**	**-6.37E-2**	**0.068**	**NA**
		HiSSE: All parameters free	0.106	0.128	0.026	0.066	0.101	2.64E-10	0.024	1.37E-10	0.001	0.128	2.80E-3	0.066	0.100
Broad	**All Species**	**HiSSE: All parameters free**	**0.530**	**0.030**	**0.117**	**0.128**	**0.058**	**0.035**	**2.65E-10**	**2.40E-10**	**1.29E-10**	**-0.005**	**0.117**	**0.128**	**0.058**
		HiSSE: No H-B, no double transitions	0.273	0.144	0.023	0.093	NA	2.98E-10	0.068	1.92E-10	NA	0.144	-0.045	0.093	NA
		HiSSE: No H-B, all transitions	0.144	0.125	0.022	0.110	NA	0.048	0.023	0.001	NA	0.077	0.000	0.109	NA
		HiSSE: No double transitions	0.053	0.026	0.137	0.087	0.248	0.017	0.059	1.79E-10	4.07E-3	0.009	0.078	0.087	0.244
	**Sympatric Species**	**HiSSE: All parameters free**	**0.630**	**0.116**	**0.020**	**0.171**	**0.052**	**2.38E-10**	**0.060**	**3.52E-8**	**0.003**	**0.116**	**-0.040**	**0.171**	**0.049**
		HiSSE: No double transitions	0.283	0.027	0.091	0.119	0.063	0.044	0.006	2.46E-10	0.027	-0.016	0.085	0.119	0.036
		HiSSE: No H-B, no double transitions	0.087	0.121	0.019	0.087	NA	2.49E-10	5.66E-2	1.80E-10	NA	0.121	-0.038	0.087	NA
	**Plethodontids**	**HiSSE: No H-B, all transitions**	**0.658**	**0.005**	**0.074**	**0.120**	**NA**	**0.016**	**1.52E-10**	**2.65E-10**	**NA**	-0.011	0.073	0.120	NA
		HiSSE: No H-B, no double transitions	0.257	0.190	0.057	0.078	NA	3.91E-10	1.17E-10	1.61E-10	NA	0.190	0.057	0.078	NA
	**Non-plethodontids**	**CID-4: Three transitions**	**0.683**	**Below**	**Below**	**Below**	**Below**	**Below**	**Below**	**Below**	**Below**	**Below**	**Below**	**Below**	**Below**
		HiSSE: No H-B, no double transitions	0.151	0.112	0.012	0.054	NA	2.31E-10	1.30E-5	1.12E-10	NA	0.112	0.011	0.054	NA
		CID-2: Three transitions, no double	0.081	0.057	0.104	0.057	0.104	0.053	0.036	0.053	0.036	0.004	0.068	0.004	0.068
		HiSSE: No double transitions	0.055	0.021	0.095	0.111	0.051	0.017	1.95E-10	0.083	0.033	0.005	0.094	0.028	0.017
		**CID-4: Three transitions**	**0.683**	*A*	*B*	*C*	*D*	*A*	*B*	*C*	*D*	*A*	*B*	*C*	*D*
				**0.097**	**0.013**	**0.079**	**0.013**	**1.99E-10**	**2.66E-11**	**1.63E-10**	**2.66E-11**	**9.67E-02**	**1.29E-02**	**7.90E-02**	**1.29E-02**

Table S10: Best fit models assuming we have sampled all species that hybridize in nature. Included are models that received >5% Akaike weights for their respective analyses. For each dataset, the best fit model is bold. Maximum-likelihood parameter estimates for speciation, extinction, and net diversification are reported. Non-hybridizing is abbreviated as NH, Hybridizing as H; A and B indicate the two hidden states. CID-4 is shown separately, as there are four hidden states included in the model, each distributed without respect to the sampled trait.

Definition of Hybridization	Dataset	Model	Akaike Weight	Speciation				Extinction				Net Diversification			
				NH-A	*NH-B*	*H-A*	*H-B*	*NH-A*	*NH-B*	*H-A*	*H-B*	*NH-A*	*NH-B*	*H-A*	*H-B*
**Narrow**	**All Species**	**HiSSE: All parameters free**	**0.518**	**0.111**	**0.030**	**0.135**	**0.064**	**2.29E-10**	**0.030**	**0.001**	**0.001**	**0.111**	**-4.00E-4**	**0.134**	**0.063**
		HiSSE: No H-B, no double transitions	0.380	0.140	0.023	0.102	NA	2.89E-10	0.068	1.51E-4	NA	0.140	-0.045	0.102	NA
		HiSSE: No double transitions	0.102	0.027	0.108	0.133	0.135	0.057	0.014	0.004	0.048	-0.030	0.094	0.129	0.087
	**Sympatric Species**	**HiSSE: No double transitions**	**0.866**	**0.086**	**0.020**	**0.312**	**0.102**	**0.004**	**0.013**	**6.43E-10**	**2.10E-10**	**0.082**	**0.007**	**0.312**	**0.102**
		HiSSE: All parameters free	0.125	0.024	0.090	0.250	0.074	0.022	1.86E-10	5.97E-10	1.53E-10	0.002	0.090	0.250	0.074
	**Plethodontids**	**HiSSE: No H-B, no double transitions**	**0.875**	**0.040**	**0.086**	**0.139**	**NA**	**0.002**	**1.77E-10**	**2.86E-10**	**NA**	**0.038**	**0.086**	**0.139**	**NA**
		HiSSE: No H-B, all transitions	0.112	0.006	0.072	0.128	NA	0.017	1.48E-10	2.65E-10	NA	-0.011	0.072	0.128	NA
	**Non-plethodontids**	**HiSSE: No double transitions**	**0.506**	**0.124**	**0.023**	**0.072**	**0.110**	**2.56E-10**	**0.018**	**0.001**	**0.004**	**0.124**	**0.005**	**0.070**	**0.106**
		CID-2: Three transitions, no double	0.383	0.083	0.014	0.083	0.014	1.71E-10	2.87E-11	1.71E-10	2.87E-11	0.083	0.014	0.083	0.014
		HiSSE: No H-B, all transitions	0.073	0.124	0.023	0.072	0.110	2.56E-10	0.018	0.001	0.004	0.124	0.005	0.070	0.106
**Broad**	**All Species**	**HiSSE: All parameters free**	**0.847**	**0.026**	**0.107**	**0.065**	**0.125**	**0.025**	**2.21E-10**	**1.33E-10**	**1.66E-4**	**0.001**	**0.107**	**0.064**	**0.125**
		HiSSE: No double transitions	0.130	0.025	0.090	0.124	0.241	0.075	0.020	0.004	4.96E-10	-0.050	0.070	0.120	0.241
	**Sympatric Species**	**HiSSE: No double transitions**	**0.932**	**0.022**	**0.067**	**0.094**	**0.193**	**0.023**	**0.007**	**0.006**	**3.98E-10**	**-0.001**	**0.060**	**0.088**	**0.193**
		HiSSE: All parameters free	0.067	0.021	0.085	0.146	0.064	0.025	1.76E-10	3.00E-10	0.009	-0.004	0.085	0.146	0.055
	**Plethodontids**	**HiSSE: No double transitions**	**0.962**	**0.016**	**0.085**	**0.117**	**0.141**	**0.048**	**1.76E-10**	**2.41E-10**	**0.006**	**-0.032**	**0.085**	**0.117**	**0.135**
	**Non-plethodontids**	**CID-2: Three transitions, no double**	**0.932**	**0.061**	**0.080**	**0.061**	**0.080**	**0.056**	**1.65E-10**	**0.056**	**1.65E-10**	**0.005**	**0.080**	**0.005**	**0.080**

Table S11: Best fit models assuming the prevalence of hybridization in nature is equal to that observed in our data. Included are models that received >5% Akaike weights for their respective analyses. For each dataset, the best fit model is bold. Maximum-likelihood parameter estimates for speciation, extinction, and net diversification are reported. Non-hybridizing is abbreviated as NH, Hybridizing as H; A and B indicate the two hidden states.

Definition of Hybridization	Dataset	Model	Akaike Weight	Speciation				Extinction				Net Diversification			
				NH-A	*NH-B*	*H-A*	*H-B*	*NH-A*	*NH-B*	*H-A*	*H-B*	*NH-A*	*NH-B*	*H-A*	*H-B*
Narrow	**All Species**	**HiSSE: No H-B, all transitions**	**0.862**	**0.018**	**0.093**	**0.146**	**NA**	**0.006**	**6.16E-4**	**0.043**	**NA**	**0.018**	**0.092**	**0.103**	**NA**
		HiSSE: No H-B, no double transitions	0.125	0.028	0.110	0.161	NA	0.029	0.001	6.23E-2	NA	0.028	1.09E-1	0.099	NA
	**Sympatric Species**	**HiSSE: No H-B, no double transitions**	**0.991**	**0.267**	**0.095**	**0.128**	**NA**	**0.032**	**0.000**	**0.023**	**NA**	**0.267**	**0.095**	**0.105**	**NA**
	**Plethodontids**	**HiSSE: No H-B, no double transitions**	**0.915**	**0.035**	**0.082**	**0.156**	**NA**	**7.28E-11**	**1.69E-10**	**3.22E-10**	**NA**	**0.035**	**0.082**	**0.156**	**NA**
	**Non-plethodontids**	**HiSSE: No double transitions**	**0.612**	**0.009**	**0.051**	**0.146**	**0.234**	**0.028**	**0.049**	**0.087**	**0.043**	**0.009**	**0.002**	**0.059**	**0.191**
		HiSSE: No H-B, no double transitions	0.298	0.097	0.012	0.098	NA	2.00E-10	0.003	0.001	NA	0.097	0.009	0.097	NA
Broad	**All Species**	**HiSSE: No double transitions**	**0.993**	**0.022**	**0.089**	**0.116**	**0.171**	**0.066**	**0.012**	**0.004**	**0.002**	**0.022**	**0.077**	**0.112**	**0.169**
	**Sympatric Species**	**HiSSE: All parameters free**	**0.999**	**0.020**	**0.090**	**0.160**	**0.102**	**0.061**	**0.001**	**0.005**	**0.003**	**0.020**	**0.088**	**0.155**	**0.099**
	**Plethodontids**	**HiSSE: No H-B, no double transitions**	**0.979**	**0.033**	**0.080**	**0.150**	**NA**	**0.000**	**3.09E-10**	**1.65E-10**	**NA**	**0.033**	**0.080**	**0.150**	**NA**
	**Non-plethodontids**	**CID-2: Three transitions, no double**	**0.958**	**0.047**	**0.080**	**0.047**	**0.080**	**0.041**	**1.65E-10**	**0.041**	**1.65E-10**	**0.047**	**0.080**	**0.006**	**0.080**

Table S12: Best fit models assuming 30% of species hybridize. Included are models that received >5% Akaike weights for their respective analyses. For each dataset, the best fit model is bold. Maximum-likelihood parameter estimates for speciation, extinction, and net diversification are reported. Non-hybridizing is abbreviated as NH, Hybridizing as H; A and B indicate the two hidden states.

Tree	Frequency of Hybridization	Dataset	Speciation λ		Extinction μ		Net Diversification $r = \lambda - \mu$		Extinction Fraction $\varepsilon = \mu \div \lambda$		Turnover $\tau = \lambda + \mu$	
			H	NH	H	NH	H	NH	H	NH	H	NH
All Species	Fully-sampled (11% N, 13% B)	N	**0.101 ± 5e-04**	0.0588 ± 1.928e-03	4.47e-10 ± 1.262e-11	**0.035 ± 7.68e-04**	**0.101 ± 5e-04**	0.0237 ± 2.68e-03	4.45e-09 ± 1.314e-10	**0.703 ± 0.0352**	0.101 ± 5e-04	0.0938 ± 1.198e-03
		B	**0.103 ± 2.22e-03**	0.0572 ± 2.02e-03	1.38e-04 ± 8.08e-06	**0.033 ± 5.52e-04**	**0.103 ± 2.22e-03**	0.0242 ± 2.56e-03	1.34e-03 ± 5.82e-05	**0.677 ± 0.032**	0.103 ± 2.24e-03	0.0903 ± 1.494e-03
	Emprical	N	**0.111 ± 1.82e-3**	0.0549 ± 1.718e-3	2.9e-3 ± 4.14e-4	**0.0335 ± 8.08e-4**	**0.108 ± 1.832e-3**	0.0214 ± 2.52e-3	0.0262 ± 3.74e-3	**0.715 ± 0.036**	**0.114 ± 1.898e-3**	0.0884 ± 9.18e-4
		B	**0.114 ± 3.46e-3**	0.0517 ± 1.97e-3	5.73e-4 ± 2.52e-5	**0.022 ± 7.36e-4**	**0.113 ± 3.46e-3**	0.0297 ± 2.7e-3	5.19e-3 ± 2.82e-4	**0.541 ± 0.0346**	**0.114 ± 3.46e-3**	0.0737 ± 1.242e-3
	20%	N	**0.124 ± 5.9e-4**	0.0552 ± 1.696e-3	9.39e-3 ± 8.62e-5	**0.0383 ± 8.36e-4**	**0.115 ± 5.04e-4**	0.017 ± 2.54e-3	0.0755 ± 3.2e-4	**0.794 ± 0.0356**	**0.134 ± 6.76e-4**	0.0935 ± 8.6e-4
		B	**0.117 ± 2.72e-3**	0.0528 ± 2.04e-3	2.39e-3 ± 1.55e-4	**0.0273 ± 7.24e-4**	**0.115 ± 2.88e-3**	0.0255 ± 2.74e-3	0.0214 ± 1.95e-3	**0.647 ± 0.039**	**0.12 ± 2.58e-3**	0.0801 ± 1.368e-3
	30%	N	**0.148 ± 9.74e-5**	0.0486 ± 1.696e-3	**0.0453 ± 5.76e-5**	6.38e-3 ± 1.85e-4	**0.102 ± 3.98e-5**	0.0422 ± 1.878e-3	0.307 ± 1.874e-4	0.164 ± 0.01032	**0.193 ± 1.55e-4**	0.055 ± 1.512e-3
		B	**0.127 ± 3.36e-3**	0.046 ± 1.73e-3	3.64e-3 ± 9.66e-5	**0.0463 ± 1.374e-3**	**0.124 ± 3.46e-3**	-3.1e-4 ± 3.1e-3	0.0293 ± 1.298e-3	**1.29 ± 0.0838**	**0.131 ± 3.26e-3**	0.0923 ± 3.56e-4
Sympatric Species	Fully-sampled (11% N, 13% B)	N	**0.0944 ± 2.96e-03**	0.0486 ± 1.632e-03	6.65e-10 ± 1.856e-11	**0.0367 ± 8.96e-04**	**0.0944 ± 2.96e-03**	0.0119 ± 2.52e-03	7.08e-09 ± 1.684e-10	**0.855 ± 0.043**	0.0944 ± 2.96e-03	0.0853 ± 7.46e-04
		B	**0.0983 ± 6.12e-03**	0.0445 ± 1.89e-03	3.61e-03 ± 7.06e-04	**0.0413 ± 1.164e-03**	**0.0947 ± 6.74e-03**	3.22e-03 ± 3.06e-03	0.052 ± 0.01222	**1.12 ± 0.069**	0.102 ± 5.5e-03	0.0859 ± 7.26e-04
	Emprical	N	**0.115 ± 9.82e-3**	0.0444 ± 1.892e-3	3.19e-5 ± 2.06e-11	**0.0105 ± 2.76e-4**	**0.115 ± 9.82e-3**	0.034 ± 2.16e-3	2.94e-4 ± 1.088e-5	**0.293 ± 0.01934**	**0.115 ± 9.82e-3**	0.0549 ± 1.616e-3
		B	**0.113 ± 6.08e-3**	0.045 ± 1.898e-3	5.03e-3 ± 4.02e-4	**0.0146 ± 6.82e-4**	**0.108 ± 6.48e-3**	0.0304 ± 2.58e-3	0.0501 ± 4.6e-3	**0.441 ± 0.0402**	**0.118 ± 5.68e-3**	0.0596 ± 1.216e-3
	20%	N	**0.111 ± 8.14e-3**	0.0419 ± 1.728e-3	8.57e-4 ± 8.68e-5	**9.45e-3 ± 3.5e-4**	**0.11 ± 8.04e-3**	0.0324 ± 2.08e-3	7.54e-3 ± 1.53e-4	**0.283 ± 0.0202**	**0.112 ± 8.22e-3**	0.0513 ± 1.378e-3
		B	**0.106 ± 4.6e-3**	0.047 ± 1.854e-3	3.02e-3 ± 1.644e-4	**0.0273 ± 1.102e-3**	**0.103 ± 4.74e-3**	0.0197 ± 2.96e-3	0.0306 ± 2.04e-3	**0.732 ± 0.0568**	**0.109 ± 4.46e-3**	0.0744 ± 7.58e-4
	30%	N	**0.128 ± 6.38e-5**	0.0407 ± 1.588e-3	0.0231 ± 4.62e-7	0.0255 ± 7.4e-4	**0.105 ± 6.42e-5**	0.0153 ± 2.32e-3	0.18 ± 9.28e-5	**0.736 ± 0.0406**	**0.151 ± 6.34e-5**	0.0662 ± 8.46e-4
		B	**0.114 ± 3.24e-3**	0.0382 ± 1.694e-3	3.53e-3 ± 1.108e-4	**0.0457 ± 1.462e-3**	**0.11 ± 3.14e-3**	-7.46e-3 ± 3.16e-3	0.0311 ± 7.14e-5	**1.48 ± 0.0972**	**0.117 ± 3.36e-3**	0.0839 ± 2.32e-4
Plethodontids	Fully-sampled (11% N, 13% B)	N	0.0687 ± 3.5e-04	0.0671 ± 2.56e-03	4.27e-04 ± 5.48e-05	2.7e-04 ± 1.002e-04	0.0682 ± 4.06e-04	0.0668 ± 2.56e-03	6.27e-03 ± 8.22e-04	4.31e-03 ± 3.8e-04	0.0691 ± 2.96e-04	0.0674 ± 2.54e-03
		B	**0.108 ± 2.44e-04**	0.0573 ± 1.342e-03	4.57e-04 ± 5.02e-05	3.28e-03 ± 2.78e-04	**0.108 ± 2.94e-04**	0.054 ± 1.586e-03	4.23e-03 ± 4.7e-04	**0.0679 ± 8.32e-03**	**0.109 ± 1.948e-04**	0.0606 ± 1.112e-03
	Emprical	N	**0.137 ± 2.72e-5**	0.0521 ± 1.524e-3	2.31e-6 ± 4.24e-7	**1.93e-3 ± 1.002e-4**	**0.137 ± 2.76e-5**	0.0502 ± 1.62e-3	1.69e-5 ± 3.1e-6	**0.0426 ± 3.08e-3**	**0.137 ± 2.7e-5**	0.0541 ± 1.43e-3
		B	**0.131 ± 2.3e-3**	0.0484 ± 2.14e-3	3.13e-3 ± 5.38e-4	**0.0245 ± 1.448e-3**	**0.128 ± 1.77e-3**	0.0239 ± 3.6e-3	0.0231 ± 3.72e-3	**0.727 ± 0.0854**	**0.134 ± 2.84e-3**	0.0729 ± 6.94e-4
	20%	N	**0.0979 ± 5.5e-6**	0.0413 ± 1.478e-3	4.26e-4 ± 1.204e-8	**0.0137 ± 7.66e-4**	**0.0975 ± 5.48e-6**	0.0276 ± 2.24e-3	4.35e-3 ± 1.63e-7	**0.435 ± 0.0512**	**0.0984 ± 5.5e-6**	0.055 ± 7.12e-4
		B	**0.143 ± 1.788e-4**	0.0491 ± 1.638e-3	3.48e-6 ± 1.334e-6	**1.4e-3 ± 6.66e-5**	**0.143 ± 1.776e-4**	0.0477 ± 1.704e-3	2.42e-5 ± 9.24e-6	**0.0332 ± 2.26e-3**	**0.143 ± 1.802e-4**	0.0505 ± 1.572e-3
	30%	N	**0.159 ± 2.08e-3**	0.0495 ± 1.532e-3	6.33e-3 ± 1.784e-3	2.23e-3 ± 3.3e-4	**0.152 ± 3.12e-4**	0.0473 ± 1.474e-3	0.0383 ± 0.01032	0.0442 ± 6.28e-3	**0.165 ± 3.86e-3**	0.0518 ± 1.654e-3
		B	**0.15 ± 1.58e-4**	0.0448 ± 1.586e-3	2.28e-5 ± 7.84e-6	**7.13e-8 ± 8.16e-9**	**0.15 ± 1.502e-4**	0.0448 ± 1.586e-3	**1.51e-4 ± 5.18e-5**	1.86e-6 ± 2.5e-7	**0.15 ± 1.658e-4**	0.0448 ± 1.586e-3
Non-Plethodontids	Fully-sampled (11% N, 13% B)	N	0.0704 ± 3.22e-04	0.0641 ± 3.28e-03	9.92e-04 ± 1.004e-05	**0.0642 ± 2.36e-03**	**0.0694 ± 3.32e-04**	-1.17e-04 ± 5.64e-03	0.0141 ± 2.06e-04	**1.15 ± 0.0902**	0.0714 ± 3.12e-04	**0.128 ± 9.4e-04**
		B	0.0703 ± 4.58e-03	0.0721 ± 3.34e-03	**6.34e-03 ± 4.54e-04**	3.58e-03 ± 1.286e-04	0.064 ± 4.76e-03	0.0685 ± 3.46e-03	0.0997 ± 0.0162	0.0614 ± 9e-03	0.0767 ± 4.44e-03	0.0757 ± 3.24e-03
	Emprical	N	0.0815 ± 3.72e-3	0.0626 ± 3.42e-3	1.16e-3 ± 2.48e-5	**7.88e-3 ± 1.07e-4**	0.0803 ± 3.72e-3	0.0547 ± 3.34e-3	0.0144 ± 5.86e-4	**0.145 ± 0.01188**	0.0826 ± 3.74e-3	0.0705 ± 3.5e-3
		B	0.0759 ± 2.12e-3	0.076 ± 1.174e-3	0.0115 ± 5.62e-3	9.68e-3 ± 3.04e-3	0.0644 ± 7.74e-3	0.0663 ± 4.2e-3	0.177 ± 0.0902	0.15 ± 0.0492	0.0874 ± 3.5e-3	0.0857 ± 1.878e-3
	20%	N	**0.0781 ± 3.38e-3**	0.0412 ± 1.734e-3	1.89e-3 ± 4.92e-5	**0.039 ± 7.88e-4**	**0.0762 ± 3.42e-3**	2.2e-3 ± 2.5e-3	0.0245 ± 1.188e-3	**1.04 ± 0.0662**	8e-2 ± 3.32e-3	0.0803 ± 9.8e-4
		B	0.0708 ± 5.42e-3	0.0719 ± 2.88e-3	1.44e-4 ± 1.038e-5	1.66e-4 ± 3.72e-7	0.0706 ± 5.42e-3	0.0717 ± 2.88e-3	2.38e-3 ± 4.8e-4	2.6e-3 ± 2.52e-4	0.0709 ± 5.42e-3	0.0721 ± 2.88e-3
	30%	N	**0.133 ± 2.46e-3**	0.0458 ± 1.806e-3	**0.0538 ± 1.12e-3**	**0.029 ± 6.06e-4**	**0.0789 ± 3.58e-3**	0.0169 ± 1.418e-3	0.407 ± 0.01384	**0.656 ± 0.0212**	**0.186 ± 1.35e-3**	0.0748 ± 2.3e-3
		B	0.0741 ± 3.52e-3	0.0735 ± 1.922e-3	8.98e-3 ± 4.44e-3	8.45e-3 ± 2.4e-3	0.0651 ± 7.98e-3	0.0651 ± 4.32e-3	0.163 ± 0.0864	0.153 ± 0.049	0.0831 ± 9.24e-4	0.082 ± 4.88e-4

Table S13: All model averaged parameter estimates. Results are reported for each diversification rate parameter quantified, for each tree, each dataset, and each assumed frequency of hybridization. Bold values indicate parameter estimates that differ significantly among character states, with the boldened values indicating the larger rate estimate. N and B correspond to the narrow and broad datasets respectively.

Tree	Frequency of Hybridization	Dataset	Probability NH > H				
			Speciation	Extinction	Net Diversification	Extinction Fraction	Turnover
All Species	Fully-sampled (11% N, 13% B)	N	0.026	1.000	0.005	1.000	0.291
		B	0.032	1.000	0.006	1.000	0.235
	Emprical	N	0.004	1.000	0.002	1.000	0.028
		B	0.012	1.000	0.003	1.000	0.035
	20%	N	0.000	1.000	0.000	1.000	0.000
		B	0.004	1.000	0.000	1.000	0.022
	30%	N	0.000	0.000	0.000	0.115	0.000
		B	0.000	1.000	0.000	1.000	0.000
Sympatric Species	Fully-sampled (11% N, 13% B)	N	0.012	1.000	0.000	1.000	0.241
		B	0.045	1.000	0.011	0.996	0.352
	Emprical	N	0.000	1.000	0.000	1.000	0.000
		B	0.000	1.000	0.000	1.000	0.000
	20%	N	0.000	0.999	0.000	1.000	0.000
		B	0.000	1.000	0.000	1.000	0.003
	30%	N	0.000	0.664	0.000	0.976	0.000
		B	0.000	1.000	0.000	1.000	0.000
Plethodontids	Fully-sampled (11% N, 13% B)	N	0.165	0.279	0.166	0.296	0.164
		B	0.000	0.949	0.000	0.975	0.000
	Emprical	N	0.000	1.000	0.000	1.000	0.000
		B	0.000	0.981	0.000	0.996	0.000
	20%	N	0.000	1.000	0.000	1.000	0.000
		B	0.000	1.000	0.000	1.000	0.000
	30%	N	0.000	0.181	0.000	0.604	0.000
		B	0.000	0.000	0.000	0.000	0.000
Non-Plethodontids	Fully-sampled (11% N, 13% B)	N	0.329	1.000	0.037	1.000	1.000
		B	0.583	0.038	0.652	0.150	0.508
	Emprical	N	0.211	1.000	0.100	1.000	0.324
		B	0.423	0.455	0.505	0.457	0.309
	20%	N	0.000	1.000	0.000	1.000	0.600
		B	0.510	0.600	0.507	0.611	0.511
	30%	N	0.000	0.000	0.000	1.000	0.000
		B	0.251	0.725	0.258	0.728	0.168

Table S14: Empirical P-values corresponding to Supplementary Table S2. Results are reported for each diversification rate parameter quantified, for each tree, each dataset, and each assumed frequency of hybridization. Empirical P-values are obtained by calculating all possible ratios between non-hybridizing (NH) and hybridizing species' (H) model-averaged parameter estimates and calculating the proportion of comparisons in which the value for the non-hybridizing lineage is greater than that of hybridizing lineage. Thus, a value of 0 means in every comparison H > NH, and vice versa. N and B correspond to the narrow and broad datasets respectively.

**Model**	**Trait-dependent Diversification**	*Best Model*	*ΔAICc < 4*	*Akaike Weights > 0.05*
BiSSE Null: One transition	No	0	0	0
BiSSE Null: Two transitions	No	0	0	0
CID-2: All transitions, all equal	No	0	0	0
CID-2: Three transitions, no double	No	0	0	0
CID-2: No double transitions, all equal	No	0	0	0
CID-4: Three transitions	No	0	0	0
CID-4: Equal transitions	No	0	0	0
BiSSE: All parameters free	Yes	0	0	0
HiSSE: No NH-B, all transitions	Yes	0	0	0
HiSSE: No NH-B, no double transitions	Yes	0	0	0
HiSSE: No H-B, all transitions	Yes	6	12	16
HiSSE: No H-B, no double transitions	Yes	21	50	53
HiSSE: All parameters free	Yes	**52**	**77**	**83**
HiSSE: No double transitions	Yes	21	36	38

Table S15: Model support for analysis of 100 phylogenies retrieved from the posterior distribution of Jetz & Pyron (2018). Values represent the number of times each model was supported according to the given condition. This set of analyses was conducted using the tree containing all species, assuming 20% of species hybridize.

Definition	Dataset	Number contrasts	P-value
Narrow	All Species	15	0.0386
	Sympatric	15	0.0156
	Plethodontids	9	0.2513
	Non-plethodontids	6	0.0166
Broad	All Species	15	0.0411
	Sympatric	18	0.0032
	Plethodontids	9	0.0735
	Non-plethodontids	8	0.0047

Table S16: Results of sister clade comparisons (Barraclough, Harvey and Nee, 1996) using each of the four datasets and two definitions of hybridization. Only sister clade comparisons with three or more taxa were considered. P-values correspond to the alternative hypothesis that species richness of clades that hybridize is greater than those that do not.

Model	Trait-dependent?	Narrow - No Glutinosus Group			Broad - No Glutinosus Group		
		AICc	Delta-AICc	Akaike Weights	AICc	Delta-AICc	Akaike Weights
BiSSE Null: One transition	No	3970.47	144.44	> 0.001	3970.55	140.81	0.000
BiSSE Null: Two transitions	No	3927.01	100.99	> 0.001	3927.20	97.46	0.000
CID-2: All transitions, all equal	No	3913.05	87.02	> 0.001	3914.49	84.75	0.000
CID-2: Three transitions, no double	No	3861.05	35.03	> 0.001	3876.95	47.21	0.000
CID-2: No double transitions, all equal	No	3902.45	76.42	> 0.001	3903.93	74.19	0.000
CID-4: Three transitions	No	3883.22	57.20	> 0.001	3879.62	49.88	0.000
CID-4: Equal transitions	No	3901.09	75.07	> 0.001	3928.09	98.34	0.000
BiSSE: All parameters free	Yes	3884.57	58.54	> 0.001	3885.26	55.52	0.000
HiSSE: No NH-B, no double transtions	Yes	3862.00	35.97	> 0.001	3863.32	33.58	0.000
HiSSE: No NH-B, all transitions	Yes	3913.07	87.05	> 0.001	3885.62	55.88	0.000
HiSSE: No H-B, no double transitions	Yes	3890.56	64.54	> 0.001	3844.78	15.03	0.001
HiSSE: No H-B, all transitions	Yes	3845.58	19.56	> 0.001	3846.27	16.53	0.000
HiSSE: All parameters free	Yes	3834.01	7.99	0.018	3835.50	5.76	0.053
**HiSSE: No double transitions**	**Yes**	**3826.02**	**0.00**	**0.982**	**3829.74**	**0.00**	**0.946**

Table S17. The 14 models fitted in our analysis excluding the *Glutinosus* group within genus *Plethodon* using both the narrow and broad definition of hybridization. Models varied in number of hidden states included as well as in the number of unique transitions among states permitted. HiSSE models contain hidden states, whereas BiSSE models do not. Double transitions are those in which two-state jumps occur (i.e. NH-**A** to H-B or vice versa). CID stands for character independent, thus CID-2 and CID-4 represent the two novel null models of trait-independent diversification that may be implemented in the HiSSE framework. Rows in bold indicate best-fit models.
